# Magnetic signatures of a creosote oil contaminated site: case study in São Paulo, Brazil

**DOI:** 10.1038/s41598-022-23493-2

**Published:** 2022-12-17

**Authors:** Carolina Silveira de Moraes, Andrea Teixeira Ustra, Alexandre Muselli Barbosa, Rosely Aparecida Liguori Imbernon, Cinthia Midory Uehara Tengan

**Affiliations:** 1 Departamento de Geofísica, Instituto de Astronomia, Geofísica e Ciências Atmosféricas, Universidade de São Paulo, São Paulo, Brazil; 2 Instituto de Pesquisas Tecnológicas, São Paulo, Brazil; 3 Departamento de Engenharia Química, Escola Politécnica, Universidade de São Paulo, São Paulo, Brazil; 4 Escola de Artes, Ciências e Humanidades, Universidade de São Paulo, São Paulo, Brazil

**Keywords:** Biogeochemistry, Environmental impact

## Abstract

Soils and groundwater contamination modifies the physical–chemical conditions of the environment, altering natural biogeochemical processes of the ground. As a result, several mineral transformations occur, in which iron plays a decisive role. The presence of iron enables the study of magnetic properties, improving the understanding of the geophysical signatures of highly dynamic environments (e.g., biogeochemical hotspots and contamination plumes). In this work, we seek to identify creosote biodegradation related to the precipitation of magnetic minerals on sediments at a contaminated site in São Paulo, Brazil. Several rock magnetism analyses were carried out to provide the magnetic mineralogy of the samples in terms of their composition, size, and abundance. We conducted high-temperature thermomagnetic curves, frequency-dependent magnetic susceptibility, anesthetic remanent magnetization (ARM) and isothermal remanent magnetization (IRM) data, superparamagnetic concentration and dipole moment (SPCDM), and scanning electron microscopy (SEM) analyses. The magnetic signatures of the contaminated samples suggest an increase of superparamagnetic grains in the water table fluctuation zone if compared to the magnetic signatures of the uncontaminated samples. Thermomagnetic curves of contaminated samples showed a lower heterogeneity of the magnetic mineral phases than the uncontaminated ones. This work contributes to the advancement of the understanding of how natural biogeochemical processes are impacted by human actions, such as soil contamination, and even by climate change, which should affect soil redox conditions in periods of drought and flooding.

## Introduction

Contamination of soils and groundwater by petroleum hydrocarbons poses a relevant risk for human health and thus it’s an environmental problem of worldwide concern. Once these chemicals are deposited on the ground, they can either be adsorbed by soil mineral phases or be leached and transported towards groundwater resources. When in contact with soils, petroleum hydrocarbons can modify the geochemical environment, affect soil microfauna and microflora, as well as natural biogeochemical cycles, and may also affect vegetation and macrofauna.

Creosote is a petroleum-derived hydrocarbon formed by the distillation of coal tar, used for preserving woods. Since creosote’s density is higher than water, it is classified as a dense non-aqueous phase liquid (DNAPL) pollutant. It means that creosote can infiltrate deeply in the soil, migrating through the saturated zone and affecting groundwater quality^1^. In the subsurface, creosote compounds will be partitioned into separate phases: air phase—contaminant present as vapors; solid phase—contaminants adsorbed or partitioned onto the soil or aquifer grain surfaces; aqueous phase—contaminants dissolved into the water according to their solubility and immiscible phase—contaminants present as dense non-aqueous liquids phase^2^. The complexity of creosote’s chemical composition imposes challenges to understanding its degradation and biodegradation processes^3^.

Biodegradation of hydrocarbons is a natural process performed by microbes dictated by the environment redox conditions, that is, terminal electron acceptors (TEAs) availability. Redox conditions are spatially and temporally variable, and evolve in terms of redox gradients, from methanogenic conditions, through sulfate, iron, manganese, and nitrate-reducing to aerobic conditions^4^.

On Earth’s surface, iron occurs in the main redox states of Fe(III) and Fe(II), being the most abundant transition metal on the Earth’s surface. Iron plays an important role in Earth’s biogeochemical cycles. For instance, contaminants strongly sorb to Fe(III) minerals and surfaces of Fe(III) (hydr)oxides also catalyze many redox transformations^5^. In fact, Fe(III) reduction has been shown to result from microbial degradation of organic compounds^6^. The authors were the first to report organisms capable of reducing the amorphic ferric oxide to extracellular magnetite during the reduction of ferric iron as the terminal electron acceptor for organic matter oxidation. Fe(III) minerals may serve as terminal electron acceptors to dissimilatory iron-reducing bacteria. The reduction of Fe(III) minerals produces soluble Fe(II) and a wide range of secondary minerals, including Fe(II), Fe(III), and mixed Fe(II)–Fe(III) minerals (Borch et al.^5^; Kappler et al.^7^ and references therein). Thus, Fe availability is a key factor for biodegradation, since Fe oxides are considered to be the dominant electron acceptor for Fe(III) reduction.

Some aerobic and anaerobic bacteria obtain energy from the oxidation of dissolved and solid-phase Fe(II) compounds to Fe(III) (oxyhydr)oxides. At circumneutral pH, aerobic oxidizing bacteria have to compete with the rapid inorganic oxidation of Fe(II) and hydrolyzation to Fe(III) (oxyhydr)oxides. These bacteria grow mainly at the low levels of oxygen typically encountered across oxic-anoxic interfaces^8^.

In biotic or abiotic processes, Fe-bearing mineral transformations are driven by the environment geochemical conditions. For example, Bodine^9^ reports pyrite and siderite formation in the sediments at a creosote-contaminated sandy aquifer in the United States, revealed by scanning electron microscopy (SEM). Iron oxide content was also higher in sediments collected from within the contamination plumes than in sediments collected from outside the plume. These iron sulfides, iron carbonates, and iron oxides, which indicate a sulfate reduction geochemical zone, could be proxied by their magnetic properties. Therefore, Environmental Magnetism is a very promising field that can provide information regarding mineral transformations, abundance, and even grain size range^10^.

Temporal changes in magnetic properties can reveal the consumption and/or the precipitation of different magnetic minerals. Lund et al.^11^ investigate the magnetic susceptibility (MS) response over time in boreholes at a hydrocarbon contaminated field research site in Bemidji, MN, USA. Redox conditions and iron availability have also been monitored at the site. The authors observed that the MS decreased over time coinciding with the depletion of solid phase iron in the source zone.

The transition from unsaturated to saturated sediments is an important redox gradient that fosters biogeochemical activity. In fact, water table (WT) fluctuations play an important role in regulating flows of carbon, nutrients, and contaminants at the watershed scale (Rezanezhad et al.^12^ and references therein). Rijal et al.^13,14^, Atekwana et al.^15^ and Beaver et al.^16^ reported large magnetic susceptibility changes in the WT fluctuation zone at hydrocarbon contaminated sites that were related to the precipitation of magnetite resulting from microbial degradation of the hydrocarbons by iron-reducing bacteria. Rijal et al.^13^ identified magnetite minerals in the single-domain range that were predominant in the WT fluctuation zone. The authors were also able to quantify these minerals and showed that the amount of newly formed magnetite increased with hydrocarbon content over time. The zone at the top of the WT seems to be the zone of strongest bacterial activity leading to iron mineralogy changes. The research conducted by Atekwana et al.^15^, and Beaver et al.^16^ at the National Crude Oil Spill Fate and Natural Attenuation Research Site administered by the United States Geological Survey (USGS) near Bemidji, MN, United States showed that the highest MS were measured in the free-phase petroleum zone around the water table, where a methanogenic community was predominant. According to the authors, this finding could be one of the first field evidence of a metabolic switching mechanism, from methanogenesis to iron reduction, with concomitant magnetite precipitation.

The respiration of iron-reducing bacteria based on solid Fe(III) mineral phases produces extracellular magnetite crystals^6,17,18^ of ultrafine-grains (about 10–50 nm). The extracellular crystallization process results in magnetic grains with superparamagnetic (SP) properties at room temperature^19^ and for this reason, this process has a unique magnetic fingerprint, recognized by the inability to sustain magnetic remanence^20^.

In this work we investigate soil cores from a creosote oil contaminated site, seeking for signs of iron-bearing mineral transformations related to the presence of the contaminants. Multiple rock magnetism techniques provided the characterization of the magnetic minerals, their relative content, and grain size range. We focus on magnetic changes observed at the WT at locations where creosote is present or absent. This work can contribute to the use of magnetic properties as proxies of the impact of DNAPLs on contaminated subsurface geochemical conditions.

### Study site

**Figure 1:**
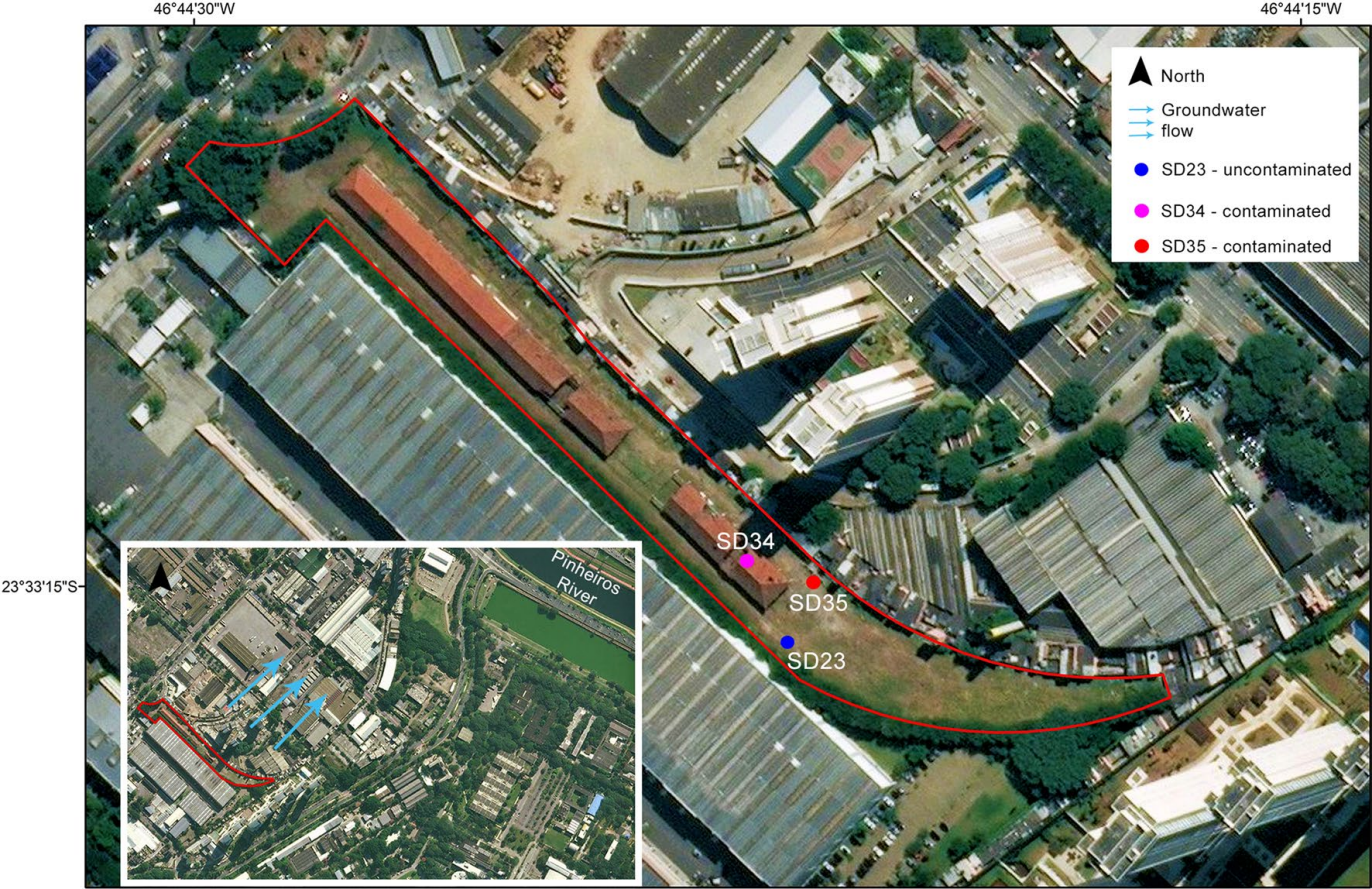
Study site with location of sampling wells and an indication of groundwater flow (blue arrows). The blue, magenta, and red dots correspond to the uncontaminated SD23 and contaminated SD34 and SD35 profiles, respectively. The inset figure shows the locations of Pinheiros river floodplain area that contributes to the subsurface stratigraphy. For the creation of the map, images obtained from the digital mapping of the city of São Paulo in 2017, available at (http://geosampa.prefeitura.sp.gov.br/), were used, and the maps were generated in the ESRI ArcMap 10.6.0 program, designed in geographic coordinate system with WGS 84.

The study site is in São Paulo, less than 1 km from Pinheiro’s River and less than 200 m from Jaguaré Creek (Fig. 1). The area is inserted in an alluvial plain and the stratigraphy produced by the constant floods is composed of layers of clay, sand, and gravel, with ascending grain sizes. These floodplain layers lie on the Tertiary deposits of the São Paulo Basin. The site is grounded by a layer of sandy clay material about 2 m thick^21^.

Between 1974 and 1997, the site served as a wood treatment plant, where chemical preservation procedures were carried out, as well the storage of sleepers used on the railroad that passed through the site. These activities involved the use of creosote oil, amongst several chemicals. The site has been studied in detail and it is currently being recovered. Aranha et al.^22^ conducted a thorough chemical characterization of the contaminants found at the site and Guireli Netto et al.^21^ investigated the contaminant distribution in the site.

## Materials and methods

### Soil samples

The Institute of Technological Research of the State of São Paulo (IPT) Waste and Contaminated Areas Laboratory (LRAC) conducted a detailed investigation at the site and confirmed the contamination of the soil and groundwater. This work investigates three soil cores acquired from this environmental investigation.

**Figure 2:**
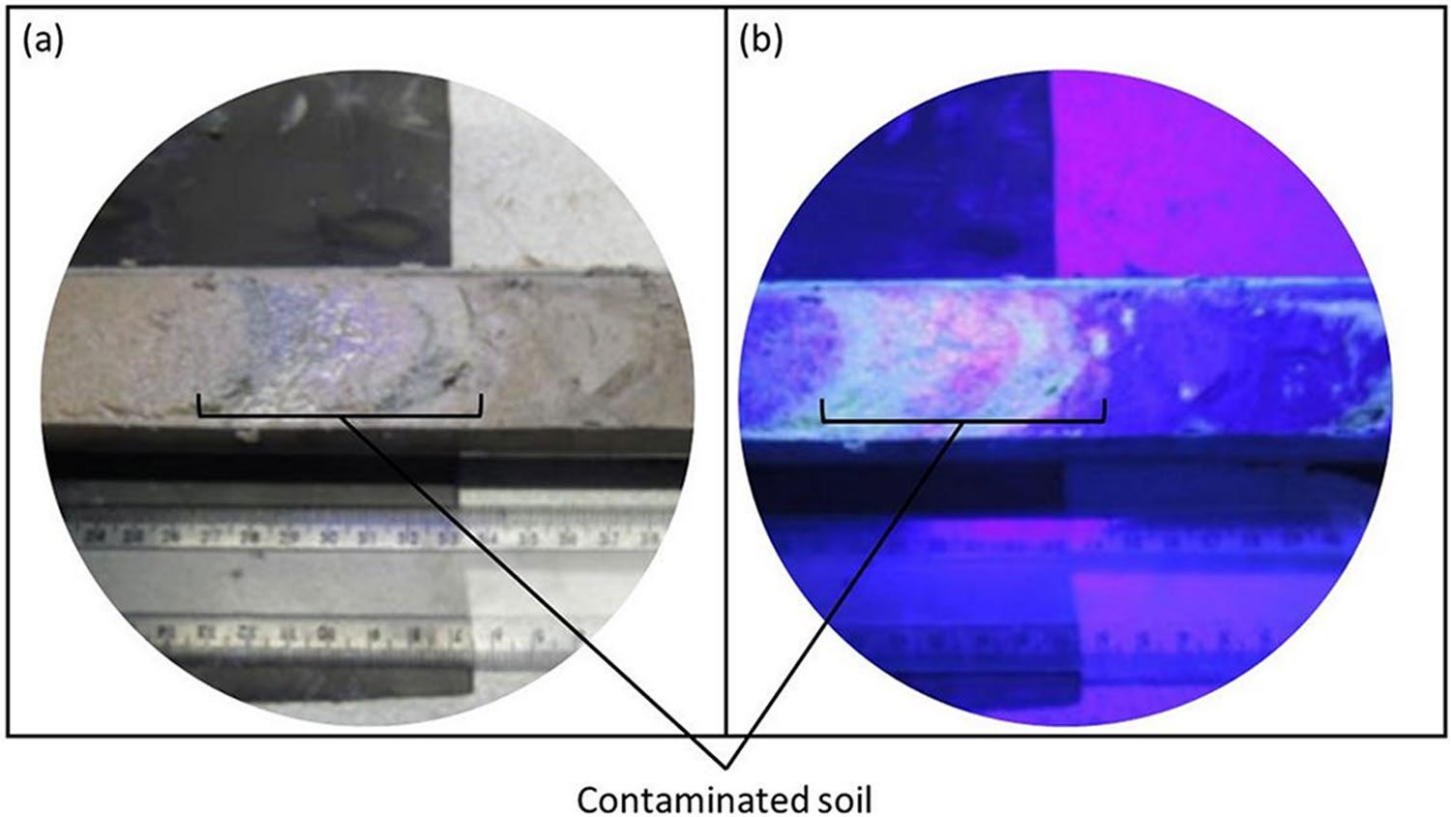
(**a**) Photograph of a section of core SD35, showing a layer of contaminated soil. (**b**) Photograph of the same core as in (**a**), showing the UV reflection by UV light of 380–420 nm (UV-A), reflection by the contaminant.

Soil core SD23 represents the uncontaminated zone in the site, assumed as the background for this work. SD 34 and 35 represent the contaminated zone since chemical contamination was detected along these two cores. 10 soil samples were collected from core SD23 and 10 from core SD34 at every 30 cm between 2.5 and 6.0 m (except to 3.3 m) along the core length. Core SD35 was also sampled from 2.5 to 6.0 m along the core, totaling 11 samples for the profile, but the sampling was guided by UV light reflection by the contaminant, as shown in Fig. 2. All samples were allowed to dry for a week at room temperature in a fume cupboard before packing them in sample holders.

### Magnetic methods

#### Thermomagnetic curves

Thermomagnetic curves record magnetic susceptibility changes as a function of temperature and are very useful to identify magnetic mineralogy by characteristic mineral transitions or alterations of mineral phase that take place at specific temperatures (e.g., Curie temperature T_C_ or Néel temperature T_N_). The Curie temperature T_C_ marks the sudden loss of magnetization when a ferri- or ferromagnetic material becomes paramagnetic in temperatures T > T_C_. The analogous for antiferromagnetic minerals such as hematite is the Néel temperature T_N_, where the mineral becomes paramagnetic at temperatures T > T_N_.

High-temperature thermomagnetic curves span the Curie and Néel temperatures of constituent minerals and show evidence of thermally induced oxidation and formation of magnetic minerals Doctor and Feinberg^23^. However, many magnetic minerals of interest in rock magnetism, such as magnetite, are altered during heating, making its identification difficult. Low-temperature protocols do not cause mineral alteration and enable the identification of specific minerals by low-temperature transitions. Hematite and magnetite can be identified by the Morin and Verwey transitions, respectively.

Soils usually contain partially oxidized magnetite grains, consisting of a maghemite surface layer and a largely unoxidized core, or even only maghemite, the fully oxidized end member of magnetite, with no Verwey transition^24^. The actual preparation for the low and high-temperature measurements favors the oxidation of the sample. For these reasons, not always magnetic mineralogy of soils is studied with low-temperature measurements.

The presence of more than one type of magnetic mineral in the matrix can make the interpretation of thermomagnetic curves a difficult task^20^. In this work, we conducted high-temperature thermomagnetic curves with Kappabridg KLY4 (AGICO) in an argon atmosphere to avoid excessive oxidation of samples during acquisition.

#### Frequency dependent susceptibility

The ability of a material to acquire magnetization per unit volume (*M*) when applied to an external magnetic field (*H*) is called volumetric magnetic susceptibility (κ)^10^. The definition of this quantity is given in Eq. (1).

1 $$\kappa = M \cdot H.$$

In this expression *M* and *H* are given in A/m and κ is a dimensionless quantity. It is still possible to obtain the mass magnetic susceptibility (χ) by normalizing κ as a function of its density (ρ), as shown in Eq. (2).

2 $$\chi = \kappa \cdot \rho .$$

Magnetic properties of soils and rocks are strongly affected by the size of the magnetic carriers. Particles in the SP-SD threshold exhibit an important magnetic fingerprint, that is the frequency dependence of magnetic susceptibility.

The SP relaxation can be observed by the loss of mass magnetic susceptibility (MS) with the increase of the external field oscillating frequency. The frequency at which MS reaches halfway of its loss is related to a characteristic relaxation time, which in turn, is related to the magnetic particle volume, according to Nèel’s theory of superparamagnetism. In this work, we carried out MS frequency-dependent measurements with MFK1 (Agico, Inc.) at three frequencies (976 Hz, 3904 Hz, and 15.616 Hz). To quantify the SP response contribution to magnetic susceptibility, the limiting frequency effect (LFE) was estimated using the procedure proposed by Ustra et al.^25^. In this procedure, a Debye relaxation model is used to fit the in-phase MS frequency measured at the MFK frequencies, yielding estimations of the low and high-frequency asymptotes, which are used to determine the LFE parameter.

#### Isothermal and anhysteretic remanent magnetization

Isothermal remanent magnetization (IRM) can be measured after exposure to a field at ambient temperature. Depending on the magnetic coercivity of the mineral and on the artificial field intensity, all magnetic moments will be magnetized in alignment with the external field. The induced magnetization does not change when increasing the applied field. This is the saturation isothermal remanence (SIRM). The anhysteretic remanent magnetization (ARM) is a stable magnetization used as a proxy for magnetite concentration. The ARM measurement involves magnetizing a sample using a low field in the presence of an alternating magnetic field that is smoothly reduced to zero.

The ARM/SIRM ratio can be analyzed as an indicator of magnetite granulometry. Higher ratio values indicate a decrease in grain size, as opposed to low values indicating an increase in the size of the magnetic population^10^. For the ARM acquisition, samples were submitted to an AF demagnetization protocol, superimposed on a constant field of 50 μT. The following field values were used: 0, 25, 30, 40, 50, 60, 70, 80, 90, 100 mT. IRM acquisition was performed, inducing magnetization in the following steps: 1000 mT and backfield 300, and 100 mT. Measurements were performed in a cryogenic magnetometer SQUID type (Superconducting Quantum Interference Device), model 755U (2G-Enterprises), and the induction by a pulse magnetizer model MMPM10 (Magnetic Measurements Ltd.), located in a shielded room at the USPMag laboratory.

#### Magnetic moment versus time

We carried out the SP Concentration and Dipole Moment (SPCDM) procedure developed by Leite et al.^26^ which essentially isolates the SP response from paramagnetic and remnant effects in the magnetic moment decay after the applied field is shut off and by the Langevin function for a magnetization curve, estimates the number of SP particles in the samples. The experimental protocol consists of the application of external fields of amplitudes ranging from 5 to 340 mT. Sample magnetization is measured during application and after removal of the external field until the equilibrium magnetization is observed. The SPCDM data were acquired with VSM Micromag 3900 (Lakeshore). According to the authors, this procedure is well suited for quantifying SP carriers with a fast magnetization decay, as expected for magnetic minerals of grain size below the SD threshold.

### Scanning electron microscopy (SEM)

Three samples were analyzed using the SEM technique. One sample from each core at the water table fluctuation zone and analyzed in terms of composition and morphology. The samples were fixed on double-sided carbon tape, specific for electron microscopy, and then received thin platinum (Pt) coating to ensure electrical conduction in Bal-tec equipment, model MED-020. They were analyzed using a Thermo Fisher Scientific scanning electron microscope, model Quanta FEG 650. The microanalyses were acquired with a Bruker EDS detector, model XFlash 6/60 with Esprit software, version 2.3.

## Results and discussion

### Magnetic mineralogy

**Figure 3:**
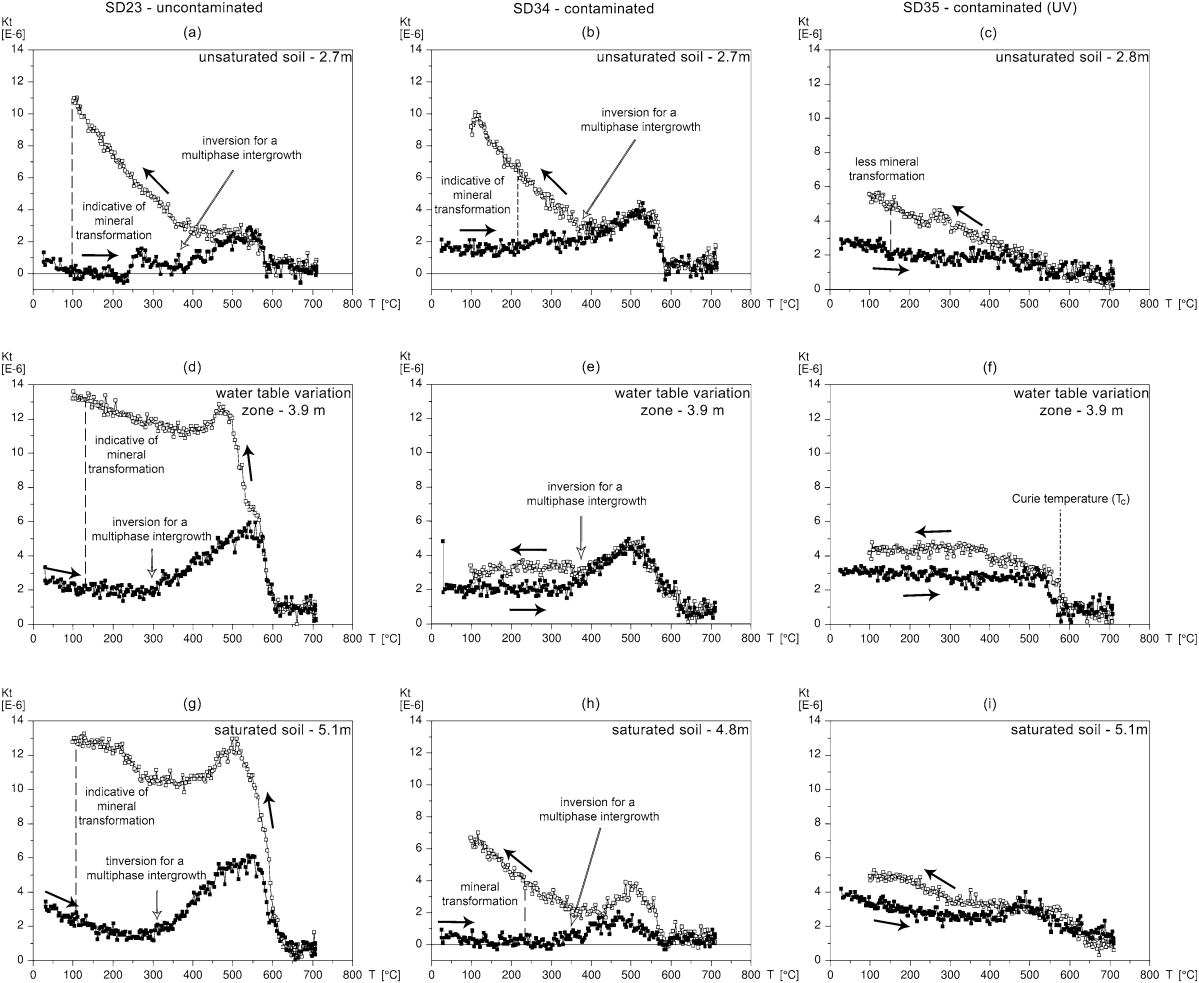
Thermomagnetic curves of uncontaminated and contaminated cores: MS changes during heating (full squares) and cooling (empty squares). (**a**–**c**) Samples from the unsaturated zone below the landfill; (**d**–**f**) Samples from the water table fluctuations zone and (**g**–**i**) Samples from the saturated zone. Arrows indicate the heating and cooling processes.

Figure 3 exhibits the variation of the magnetic susceptibility in the function of the temperature. To the contaminated profiles (SD34 and SD35), heating and cooling curves do not present high variations, unlike the SD23 profile. Figure 3a shows transformations between 200 and 350 °C, suggesting the presence of iron hydroxides, such as ferrihydrite, lepidocrocite, and goethite, typically forming in soils (Hansech et al.^27^ and references therein). This variation between heating and cooling curves indicates the occurrence of mineral transformations. The smallest occurrence of mineral transformations in the contaminated profiles can be explain as an impact of the creosote oil in the soil. The aromatic rings that makeup creosote tend to be adsorbed on the solid particles of the sediments, due to their hydrophobicity and low water solubility^28^. That can significantly modify the availability or access to iron, hindering the biotic or abiotic electron transfer process and inhibiting or limiting mineral transformations in the natural soil, which reflects in a poor diversity in contaminated samples.

The curves of the SD23 (uncontaminated) and SD34 (contaminated) profiles present a similar behavior characterized by an increase in susceptibility between about 300 and 550 °C and a sharp drop at about 580 °C. The result is similar to that present by Özdemir and O’Reilly^29^ for synthetic monodomain titanomaghemite, indicating that this may be a mineral present. The presence of titanomagnetite/titanomaghemite is mainly due to the ubiquitous occurrence in the sediments transported along the aluvionar environment, and to the regional geology and granite-gneiss embasement, which often contains titanomagnetite can also contribute to the iron minerals source.

The high-temperature oxidation process (deuteric oxidation) of titanomagnetites and titanohematites results in the intergrowth of a titanium-rich phase (ilmenite) and an iron-rich phase (which can be either magnetite or hematite). Özdemir and O’Reilly^29^ discuss that any experiment involving the heating of titanomaghemites to temperatures above about 350 °C is characterized by inversion (analogous to the hematite-maghemite inversion) for a multiphase intergrowth. Thus, the increase in susceptibility in the heating curves at 350 °C in the thermomagnetic curve of the SD23 and SD34 cores are a result of this mineral inversion, while the increase in susceptibility observed during cooling (which makes the curve irreversible) may be a result of this mineral inversion, a product of the deuteric oxidation process that forms ilmenite and magnetite^27^. In that sense the curves indicate the absence or a significantly less amount of titanomaghemite in the contaminated samples. This depletion may be a consequence of an anoxic environment, preventing the oxidation of titanomagnetite to titanomaghemite, while the uncontaminated samples undergo oxidation.

The mineral transformations we see on the thermomagnetic curves of contaminated samples are distinct from transformations we see on the uncontaminated samples because the geochemical environment, and therefore the mineral phases, are distinct. For example, Hansech et al.^27^ show that the presence of organic matter causes critical changes in the reaction of iron minerals during heating. Further investigation of the contaminated composition may explain the transformation inhibition of the transformations during heating.

**Figure 4:**
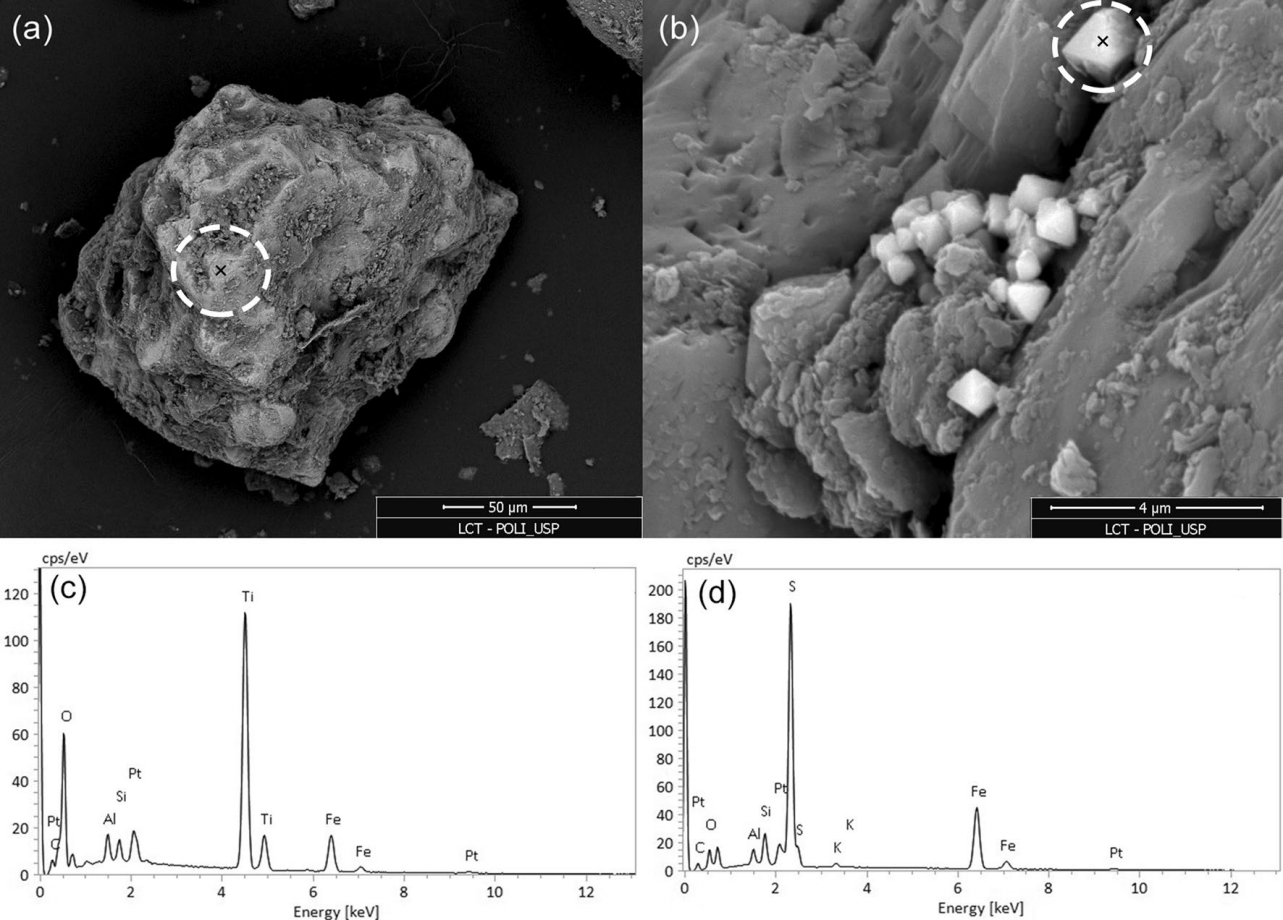
SEM image of sample from the contaminated zone at the water table fluctuation zone (**a**) SD 35 at 3.88 m and (**c**) SD34 at 3.9 m. The EDS analysis of (**a**) corresponds to the octahedral prism-shaped grains seen in (**b**), which was identified as an iron sulfide mineral, and the EDS analysis presented in (**c**) corresponds to where iron–titanium is identified (**d**). The “X” inside the circles indicates the location of the EDS analysis.

The SEM images presented in Fig. 4 identified iron–titanium oxides in the SD34 analysis, supporting the thermomagnetic analysis. The SEM results show iron sulfides in the SD35 sample. Even though there is no sign of iron sulfides in the thermomagnetic analysis in Fig. 3f, it must be clear that the samples in the two analyses were not the same but from the same depth. Iron sulfides, on the other hand, are indicative of biogeochemical pyritization due to the reducing environment. That may explain why we do not observe oxidation in thermomagnetic analysis in the contaminated region, while the uncontaminated samples undergo oxidation. The identification of pyrite by SEM strongly supports the modification of the contaminated location to a reducing environment, in agreement with the maghemite depletion detected by the thermomagnetic curves.

### Magnetic mineralogy and grain size effects

**Figure 5:**
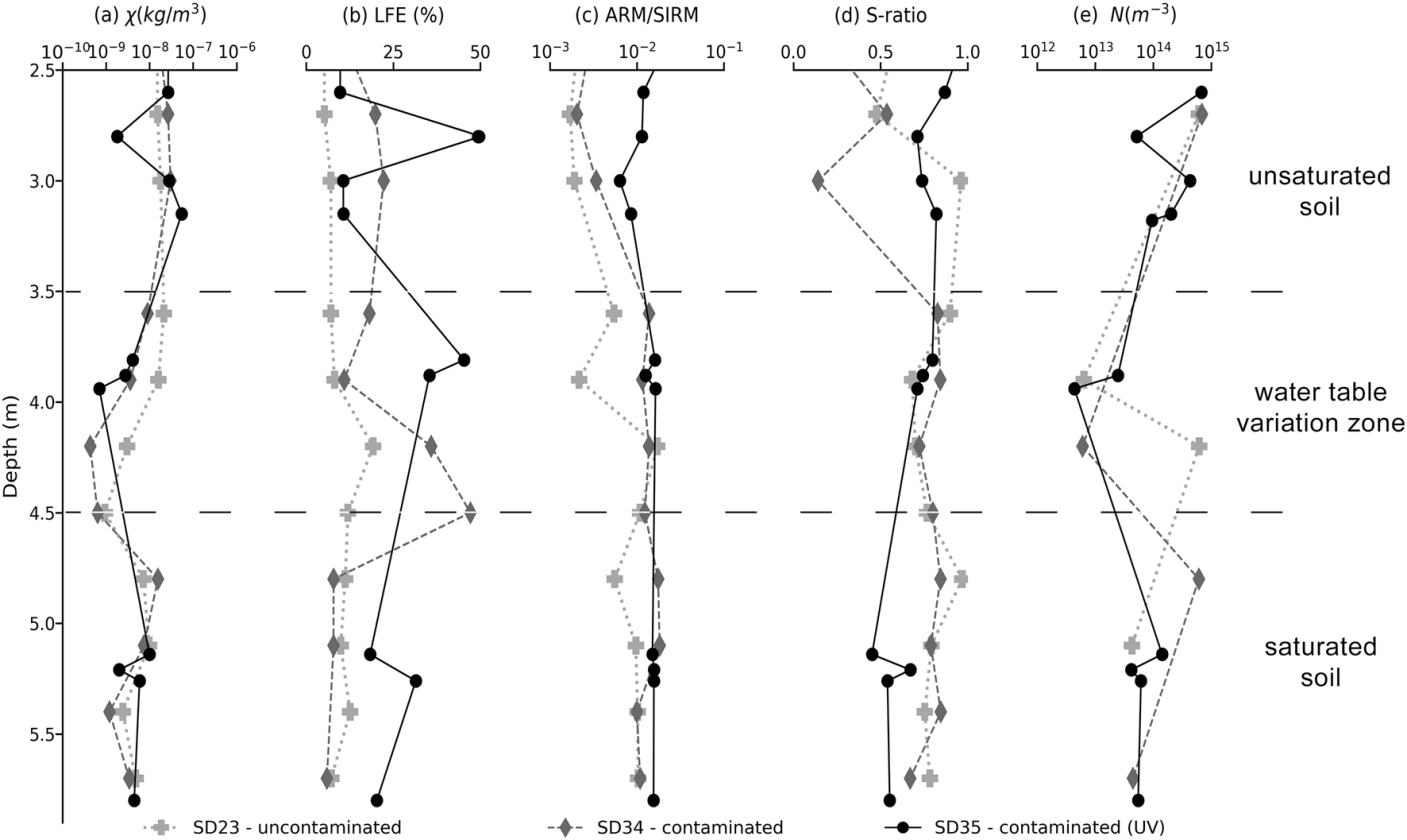
Magnetic properties depth profiles of SD23 (uncontaminated), SD34 and SD35 (contaminated) boreholes. (**a**) MS at the frequency 976 Hz; (**b**) Frequency effect represented by the LFE parameter; (**c**) ARM/SIRM ratio; (**d**) S-ratio and (**e**) number of SP grains. While (**a**) shows how magnetic the samples are, (**b**) and (**c**) reveal the magnetic signature of the finer magnetic carriers in the samples, (**d**) reveals the occurrence of magnetic minerals of different coercivities and (**e**) gives a quantification of the ultrafine content.

The multi-frequency magnetic susceptibility values obtained at 976 Hz are exhibited in Fig. 5a. The three profiles are characterized by maximum values of MS followed by a minimum value at the water table variation zone at about 4.25. For the uncontaminated profile (SD23), the maximum MS value corresponds to 20^10^ − 8 m^3^/kg being approximately constant between 2.50 and 4.00 m. On the other hand, the contaminated profiles SD34 and SD35 do not present a maximum constant value of MS. The SD34 profile shows a MS peak of 15^10^–7 m^3^/kg at 3.15 m depth, and the SD35 profile shows a peak of 75^10^–7 m^3^/kg at 4.00 m depth.

Higher variations at the water table transition zone are accordant with Atekwana et al.^15^ observations, which show peaks of magnetic susceptibility at the interface of the unsaturated and saturated zones for a region contaminated by hydrocarbons. The result suggests the occurrence of ferromagnetic minerals such as magnetite, which has a high susceptibility, or the increase in the concentration of particles, which can occur because of microbial activity. According to the authors, the MS increase within these depths suggests that this interval may have optimum water saturation conditions necessary for producing maximum rates of biodegradation, that is, a biogeochemical hotspot. However, in our study, MS was not enhanced, and its profile doesn’t allow making obvious distinctions between contaminated and uncontaminated samples. We believe that, since MS reflects mineral phase, abundance, and grain size of magnetic grains, it is sensitive to most transformations is biogeochemical.

Figure 5b exhibits the values of the LFE parameter. The value for SD23 is approximately constant, not exceeding 15%. On the other hand, the SD34 and SD35 profiles present peaks that reach values higher up to 50%. Maximum parameter occurs at the interface of the saturated zone for both profiles. For the SD34 profile, the maximum LFE parameter value occurs at 4.50 m and for the SD35 profile at 2.50 m and 3.80 m. It’s important to highlight that depths above 2.50 m correspond to a clayey landfill in the studied location. This analysis makes it possible to distinguish between contaminated and uncontaminated samples. Figure 5c shows the ARM/SIRM environmental parameter. This parameter can indicate the decrease in the size of magnetic particles by higher parameter values. In the figure, values of the contaminated (SD34 and SD35) are higher and approximately constant, revealing the magnetic values of smaller granulometry or concentration compared to the background profile (SD23). Figure 5d presents the S-ratio which higher values of this ratio indicate a major contribution of soft minerals to magnetic susceptibility. The graph presents values close to 1 for all profiles, indicating the predominance of soft minerals. The concentration of SP particles is exhibited in Fig. 5e. The graph indicates the decrease in the concentration of SP particles for the three profiles around 4.00 m at the water table. At first, it seems that this result contradicts the results obtained in Fig. 5b, but this does not occur. The LFE parameter can identify SP-SSD size range particles, and the SPCDM procedure analyzes strictly SP particles, which present relaxation times not captured by the frequency dependence MS instrumentation used in this study.

The parameters affected by the grain size allowed us to identify the magnetic response of particles in the grain size range of the SP-SSD domains. This response can be interpreted as signs of oil biodegradation since hydrocarbons provide a source of carbon to bacteria and stimulate microbial growth and their metabolic byproducts. For instance, Fe-reducing bacteria can increase ultrafine magnetite particles (SP-SSD) in the medium due to their metabolic activity. The S-ratio parameter also agrees with the biodegradation hypothesis because higher contributions of soft minerals indicate that magnetite may be the magnetic mineral responsible for the increase of magnetic susceptibility. Rijal et al.^13^ also observed the increase of SP–SD magnetite concentration parameters in this dynamic region. The increased susceptibility in the water level zone (for the contaminated sample) is in good agreement with extensive literature involving soils, contaminated land, and biogeochemical processes, which report the increase of magnetite within this zone. These new minerals are mainly magnetite nanoparticles that exhibit superparamagnetic properties, that is, high magnetic susceptibility and frequency-dependent susceptibility.

The MS profiles, against LFE and the other environmental parameters, reveal the coupled abiotic-biotic pathways to iron transformations. Reductive dissolution of iron (oxi)hydroxides present in the natural soils may have been enhanced in the contaminated location. However, according to Hansel et al.^30,31^, at high Fe(II) concentrations, secondary mineralization involves an interplay between geochemical factors and competing mineralization processes. In the contaminated region, magnetite may be further precipitated, fostered by microbial activity, at the expense of other Fe-minerals precipitation, resulting in ultrafine magnetite (higher LFE) and low mineral diversity.

## Conclusion

Biogeochemical cycling of iron is crucial to the fate of contaminants in soils and groundwater and therefore plays an important role on water and food security. Changes in the redox conditions of the environment are determinant conditions for a complex network of biogeochemical interactions that dictates the speciation, mobility and reactivity of iron in the environment. We have presented a careful and extensive analysis of magnetic properties of sediments retrieved from a creosote’s contaminated site in Brazil. The results obtained provide some important insights regarding the magnetic mineralogy and grain sizes changes, which can be related to the contaminant impact on the geochemical environment.

The magnetic mineralogy of the contaminated sample is indicative of a reducing environment, where pyrite is formed and iron oxides are transformed. The size-related magnetic parameters obtained from different methods agree to suggest that the contaminated samples presented a stronger superparamagnetic signature, which indicates the presence of ultrafine magnetic particles, the expected size range of biogenic extracellular Fe-bearing minerals. The magnetic properties of samples from both locations exhibit highest variations around the water table fluctuation zone, a known biogeochemical hotspot.

## Supplementary Information

Supplementary Information 1.

Supplementary Information 2.

Supplementary Information 3.

## Data Availability

The datasets used and/or analysed during the current study available from the corresponding author on reasonable request.
