# Supplementary figures and images for: Magnetic signatures of a creosote oil contaminated site: case study in São Paulo, Brazil
Source: Sci Rep. 2022 Dec 17;12:21853. doi: 10.1038/s41598-022-23493-2 (PMC9759522; doi:10.1038/s41598-022-23493-2)

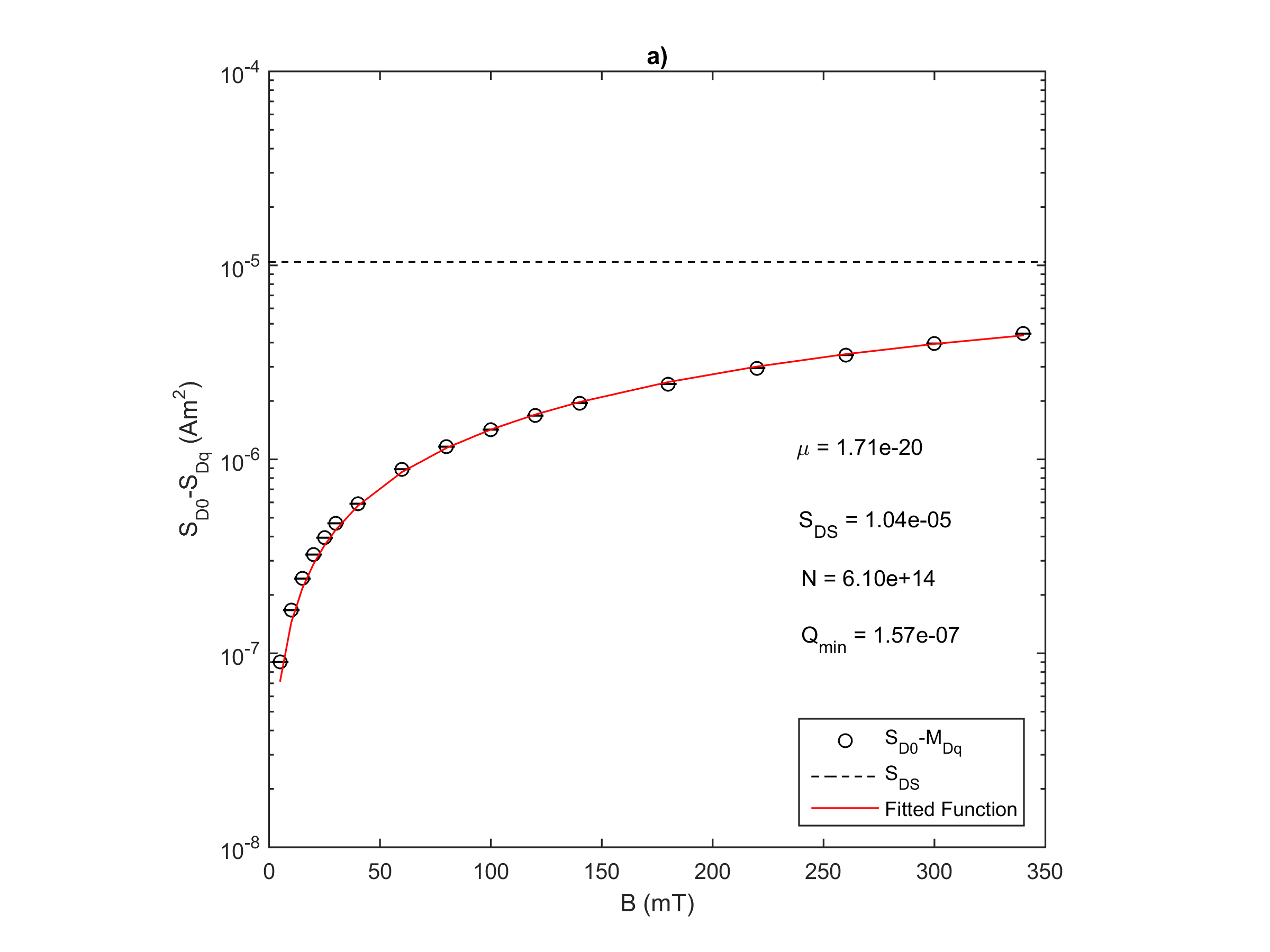

Supplement: Supplementary file 3 — Supplementary Information 3. [file 41598_2022_23493_MOESM3_ESM.zip › moment_vs_time/SD23/2_7/nm1xxx/ajuste.png]

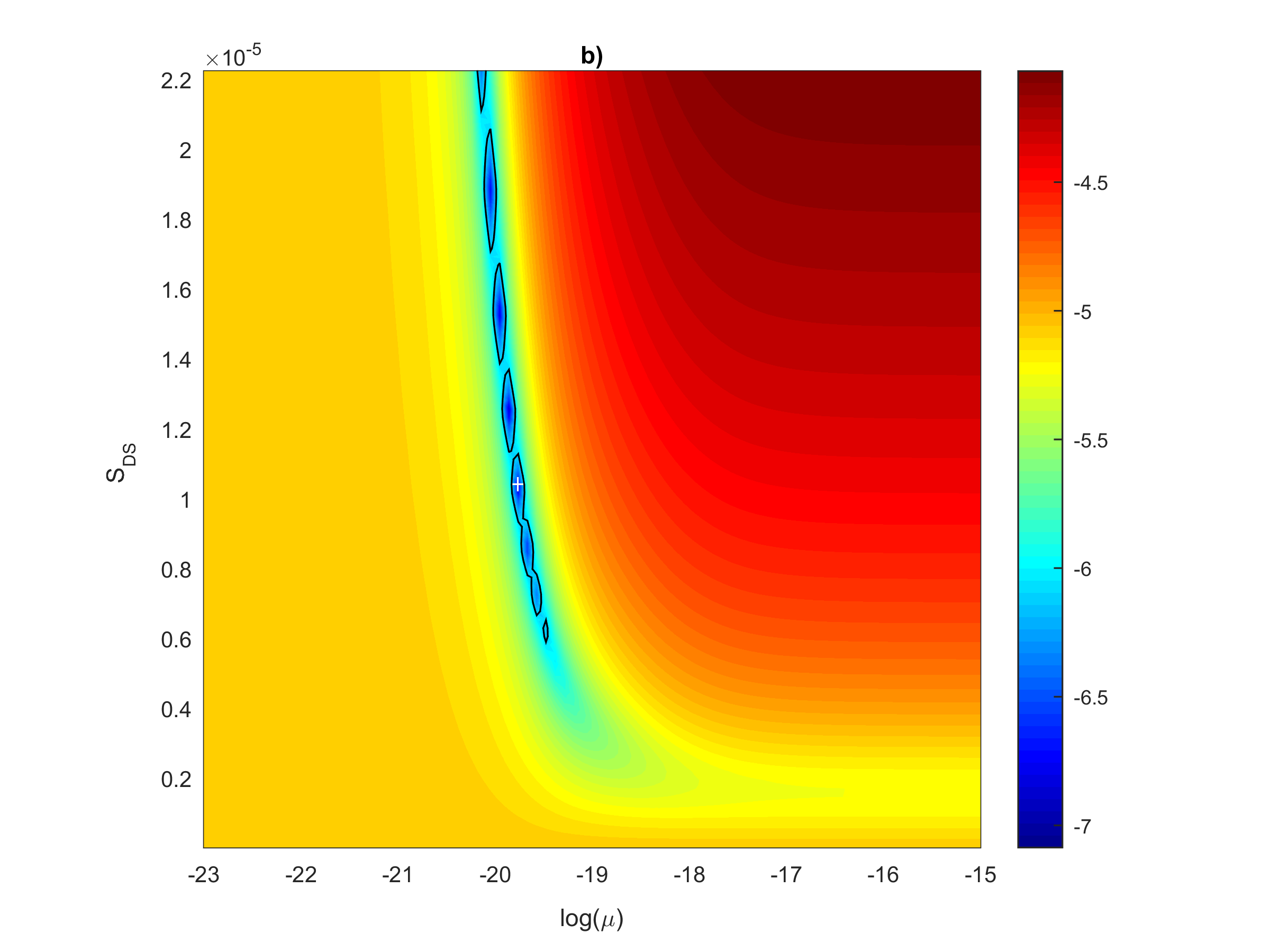

Supplement: Supplementary file 3 — Supplementary Information 3. [file 41598_2022_23493_MOESM3_ESM.zip › moment_vs_time/SD23/2_7/nm1xxx/mapa.png]

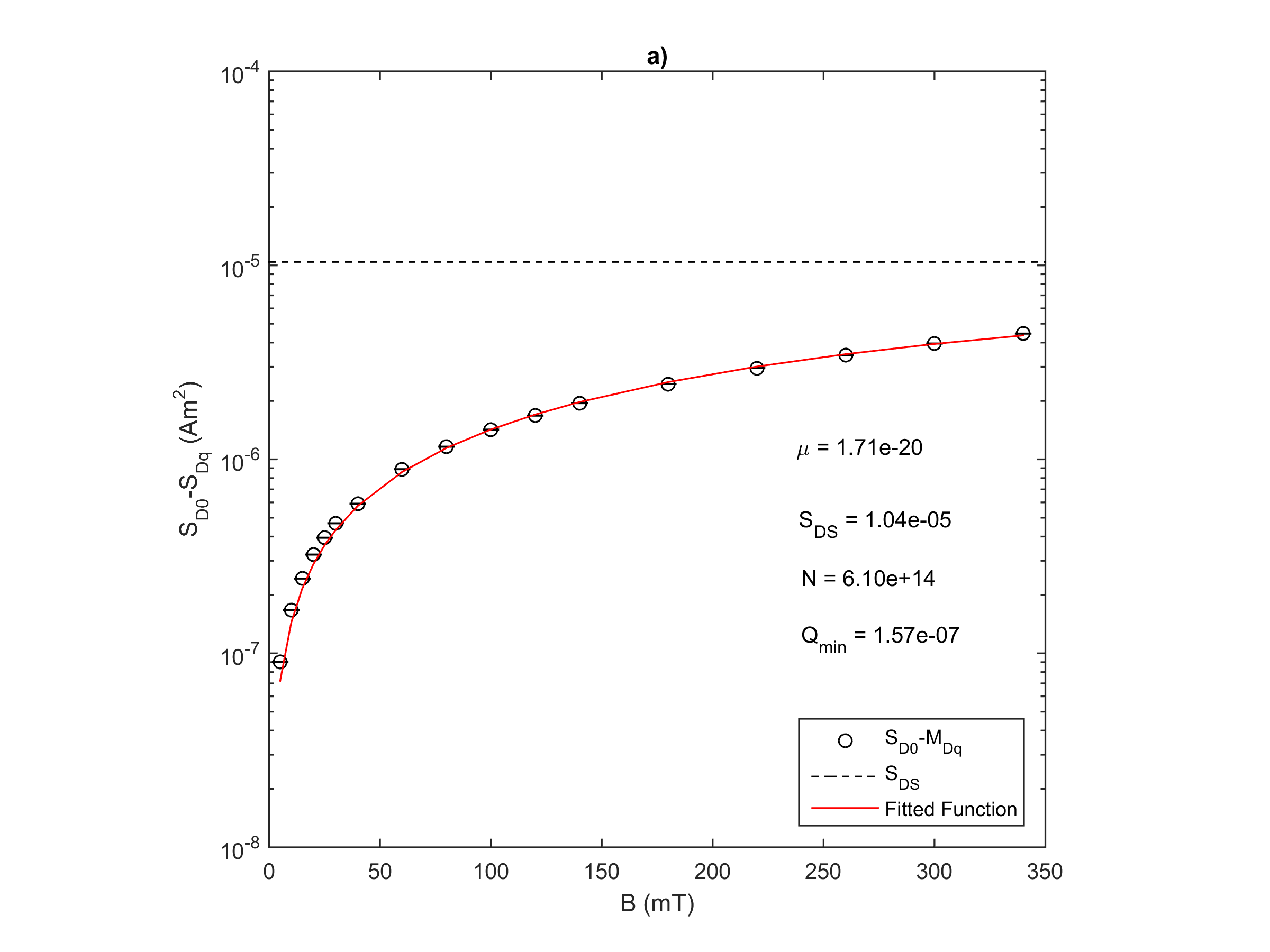

Supplement: Supplementary file 3 — Supplementary Information 3. [file 41598_2022_23493_MOESM3_ESM.zip › moment_vs_time/SD23/2_7/nm1xxx/p2.tif]

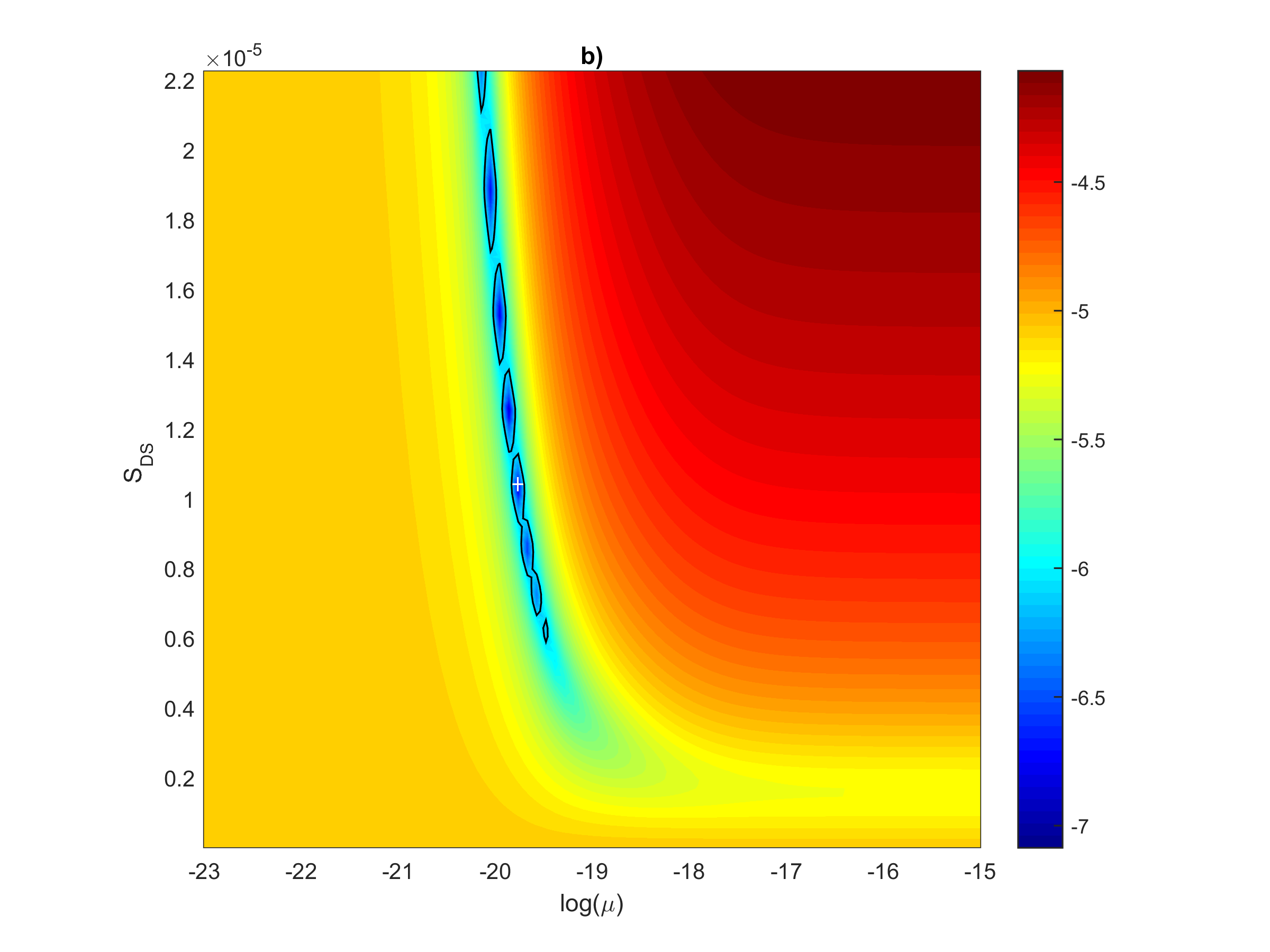

Supplement: Supplementary file 3 — Supplementary Information 3. [file 41598_2022_23493_MOESM3_ESM.zip › moment_vs_time/SD23/2_7/nm1xxx/p3.tif]

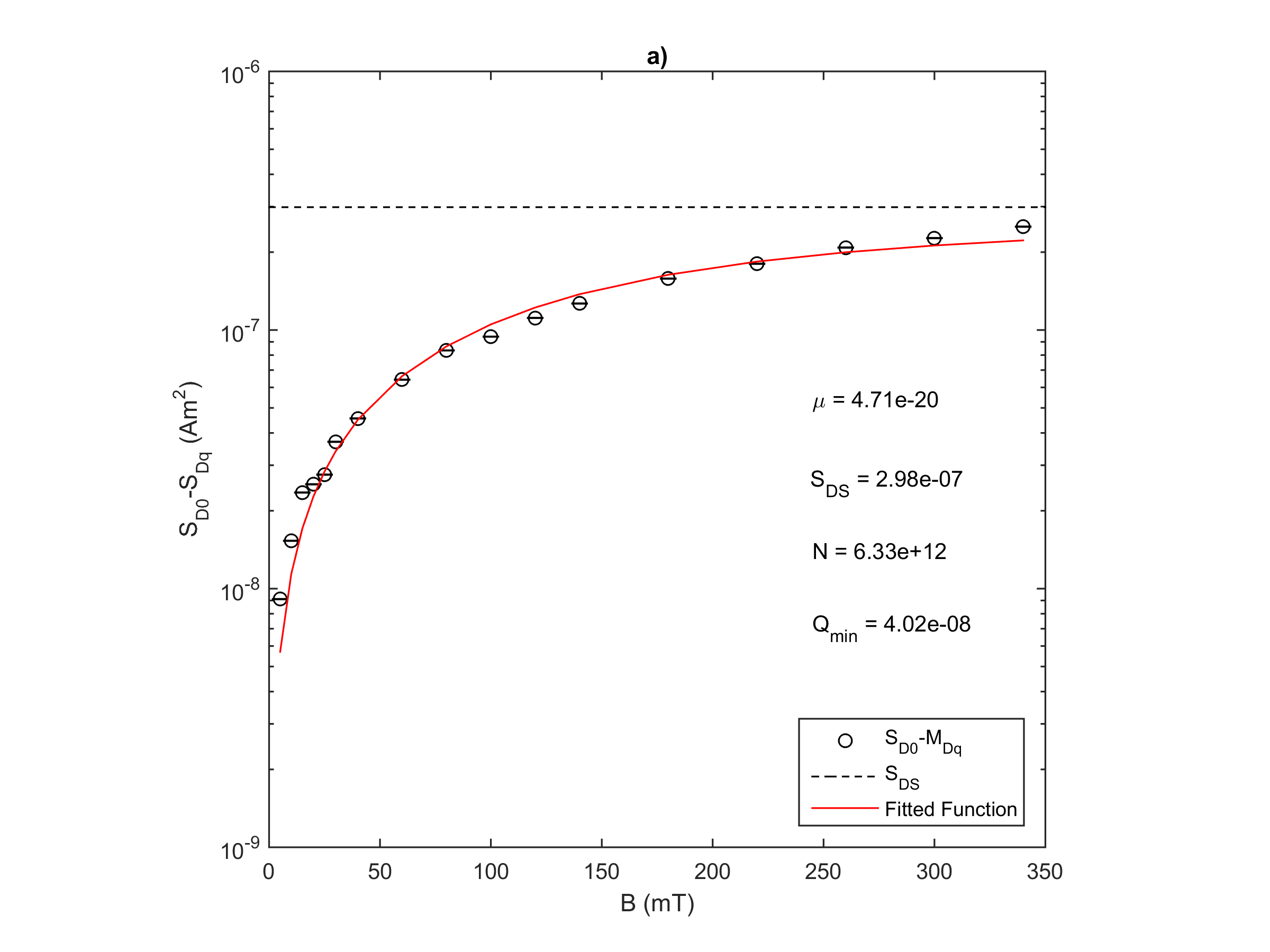

Supplement: Supplementary file 3 — Supplementary Information 3. [file 41598_2022_23493_MOESM3_ESM.zip › moment_vs_time/SD23/3_9/nm2xxx/ajuste.png]

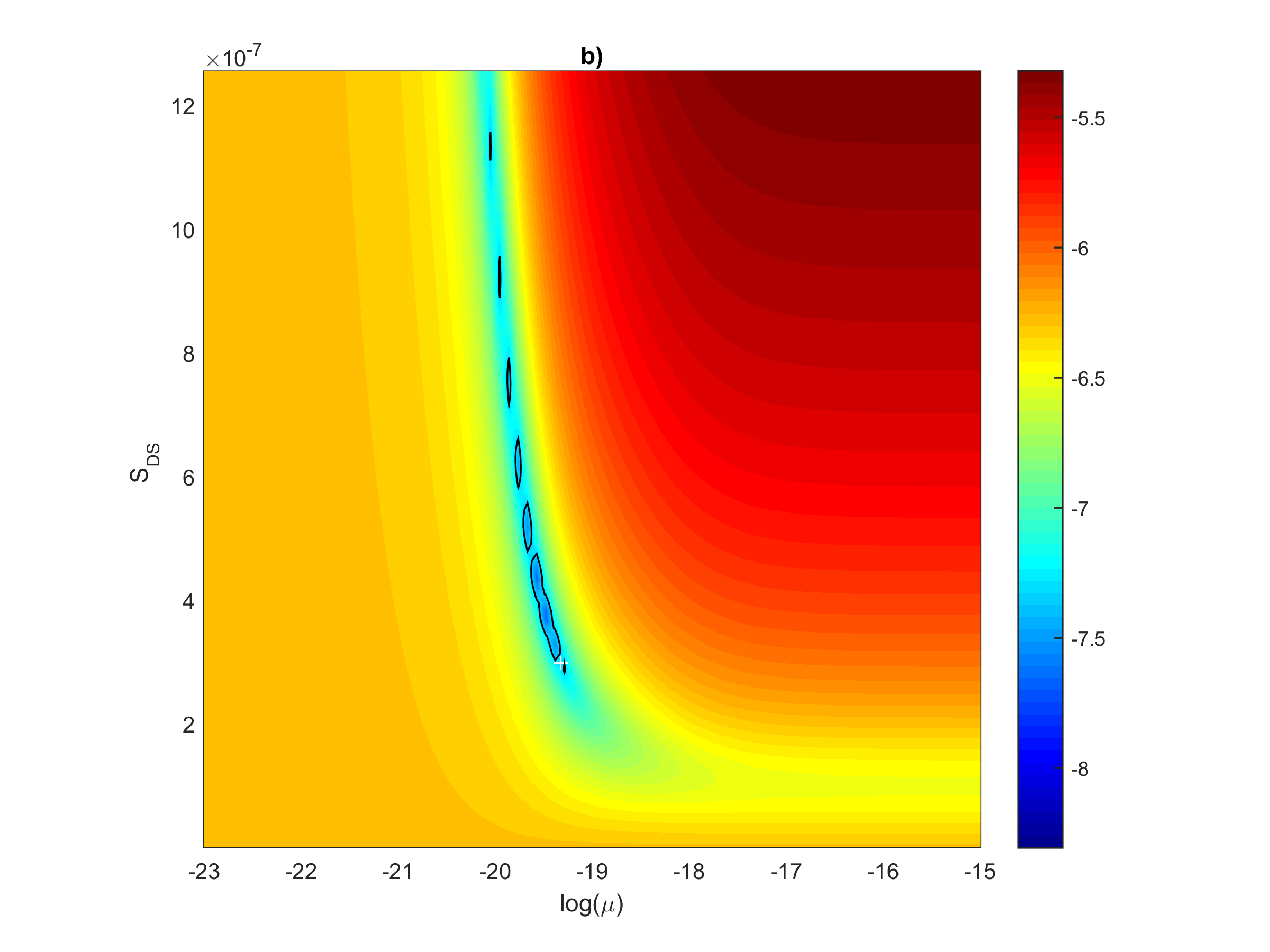

Supplement: Supplementary file 3 — Supplementary Information 3. [file 41598_2022_23493_MOESM3_ESM.zip › moment_vs_time/SD23/3_9/nm2xxx/mapa.png]

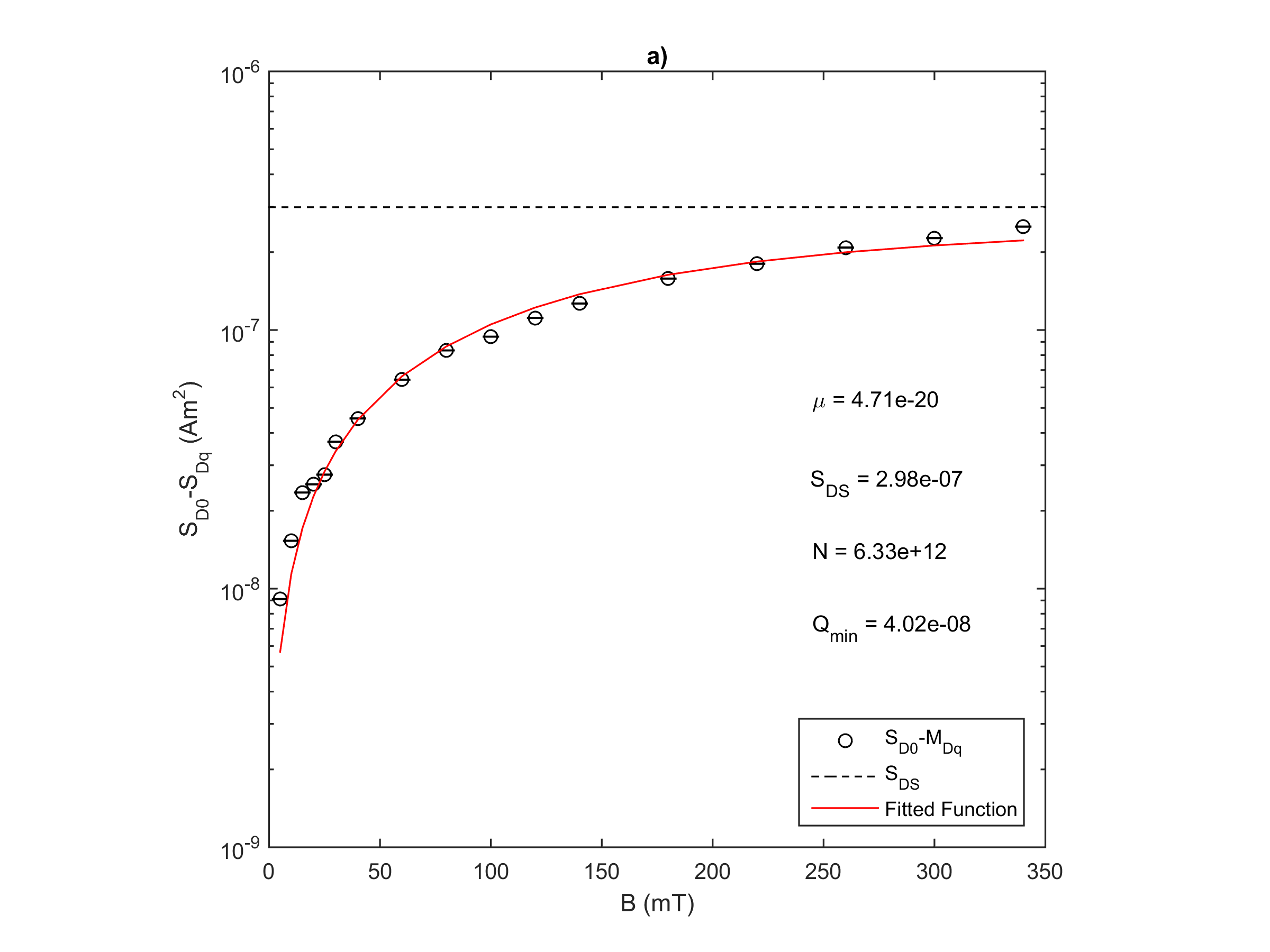

Supplement: Supplementary file 3 — Supplementary Information 3. [file 41598_2022_23493_MOESM3_ESM.zip › moment_vs_time/SD23/3_9/nm2xxx/p2.tif]

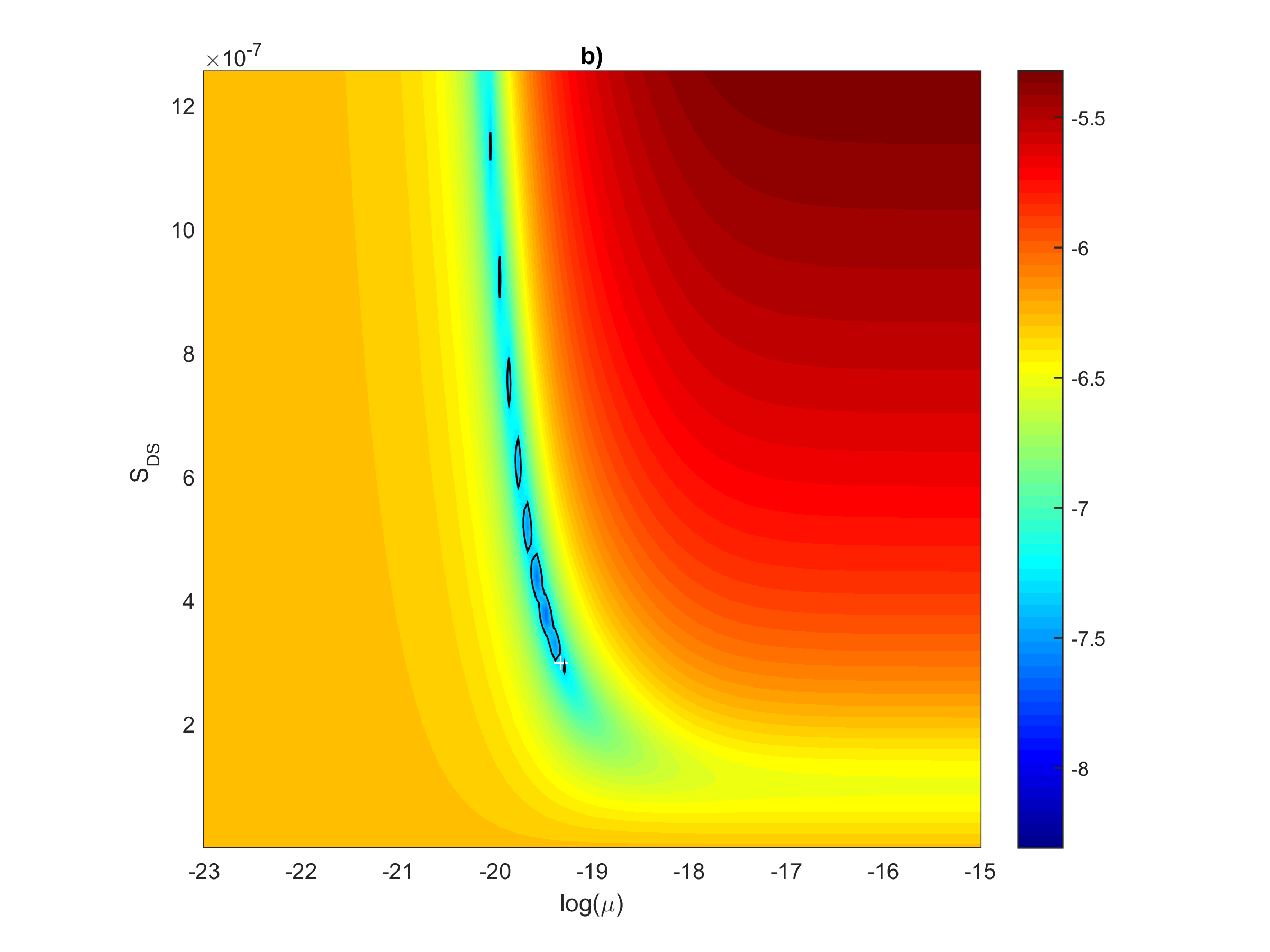

Supplement: Supplementary file 3 — Supplementary Information 3. [file 41598_2022_23493_MOESM3_ESM.zip › moment_vs_time/SD23/3_9/nm2xxx/p3.tif]

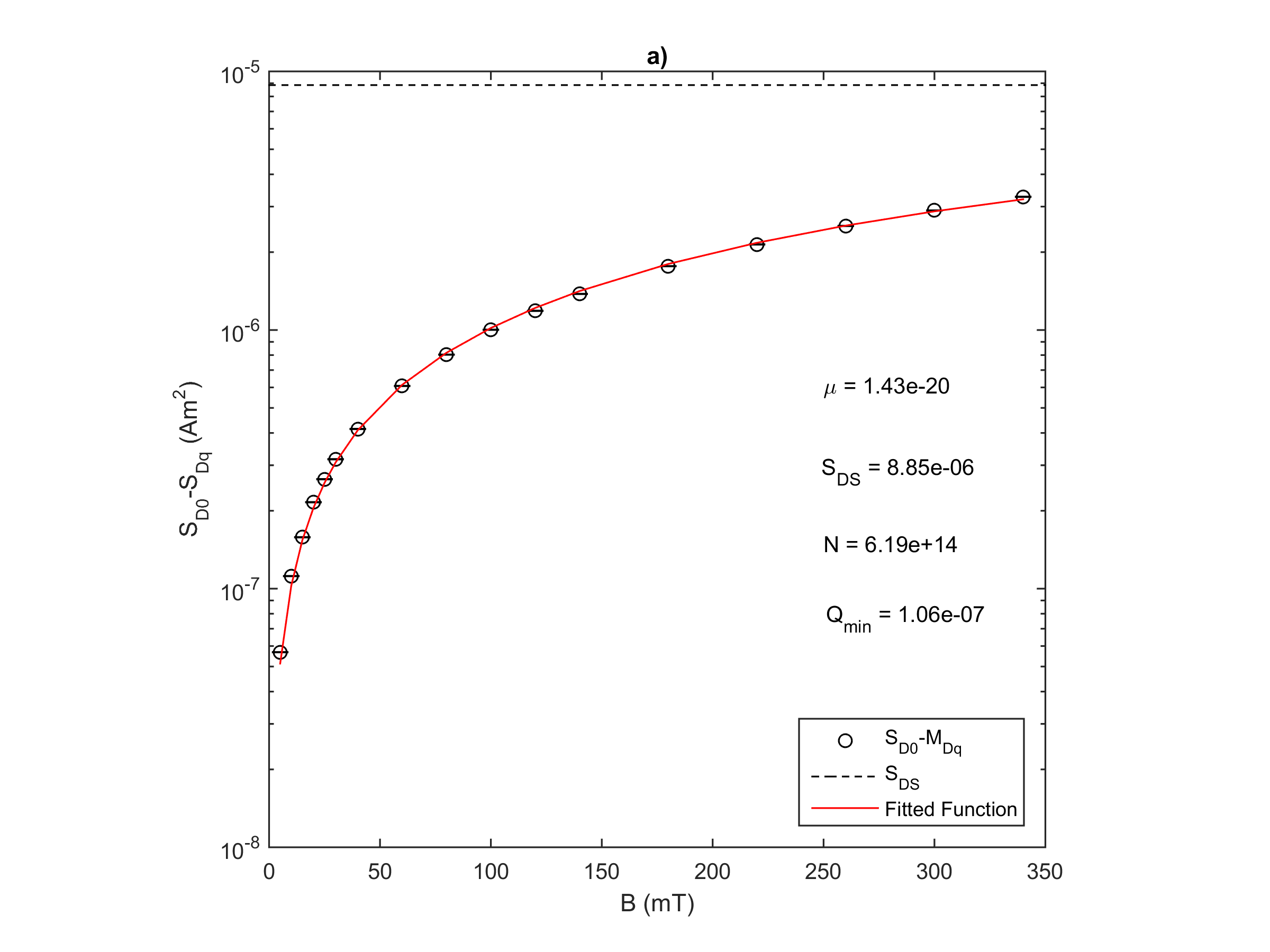

Supplement: Supplementary file 3 — Supplementary Information 3. [file 41598_2022_23493_MOESM3_ESM.zip › moment_vs_time/SD23/4_2/nm3xxx/ajuste.png]

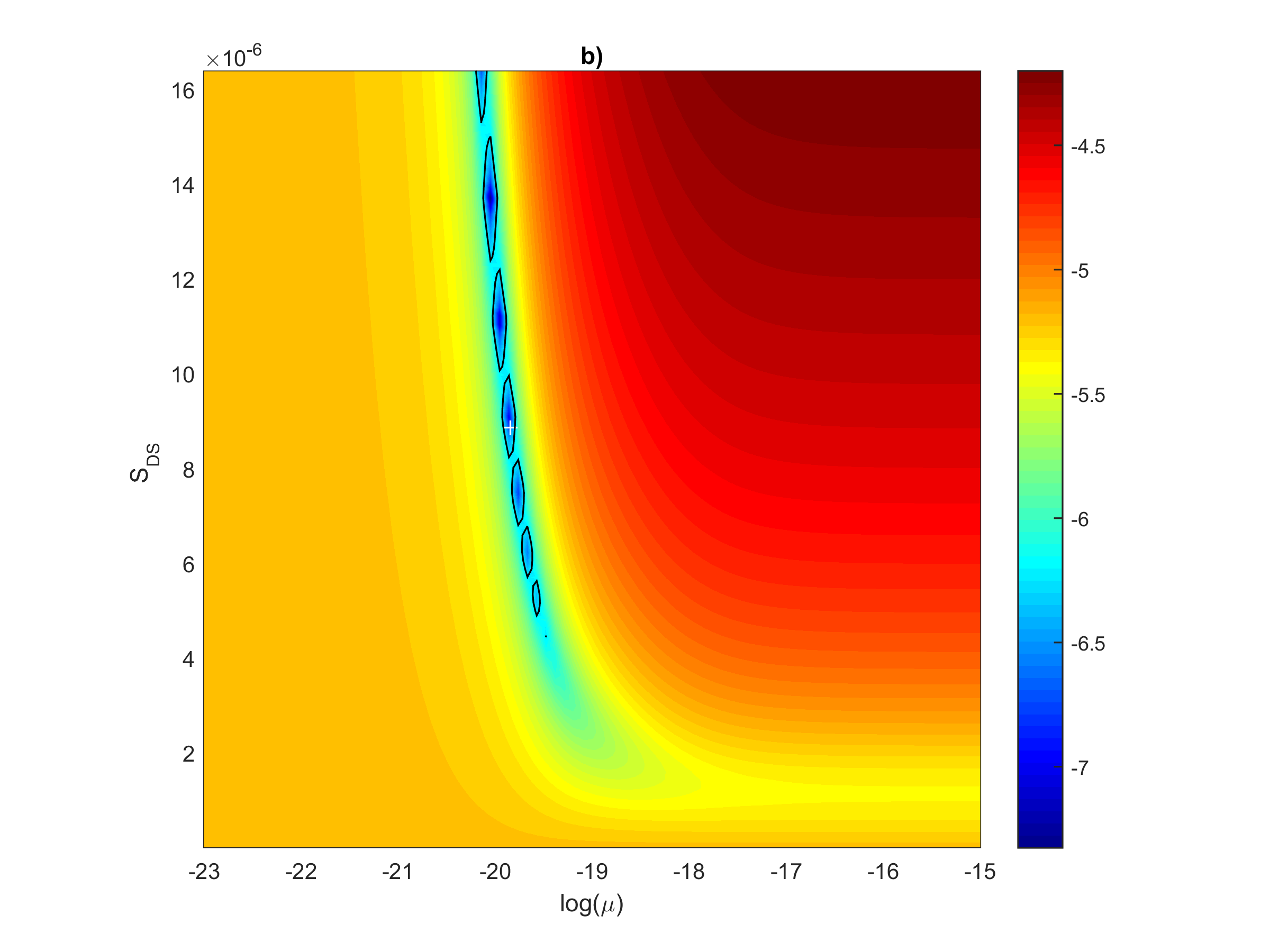

Supplement: Supplementary file 3 — Supplementary Information 3. [file 41598_2022_23493_MOESM3_ESM.zip › moment_vs_time/SD23/4_2/nm3xxx/mapa.png]

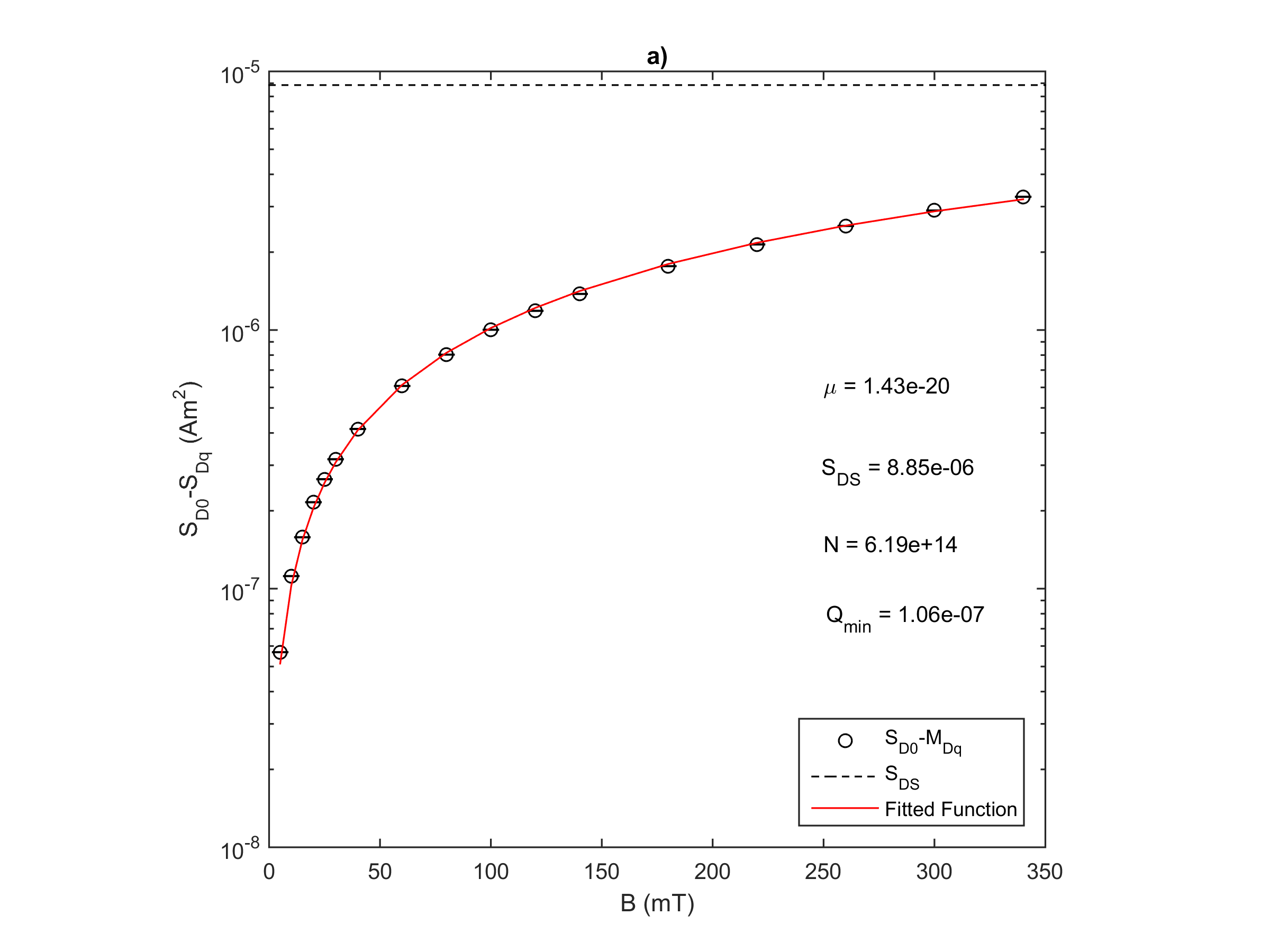

Supplement: Supplementary file 3 — Supplementary Information 3. [file 41598_2022_23493_MOESM3_ESM.zip › moment_vs_time/SD23/4_2/nm3xxx/p2.tif]

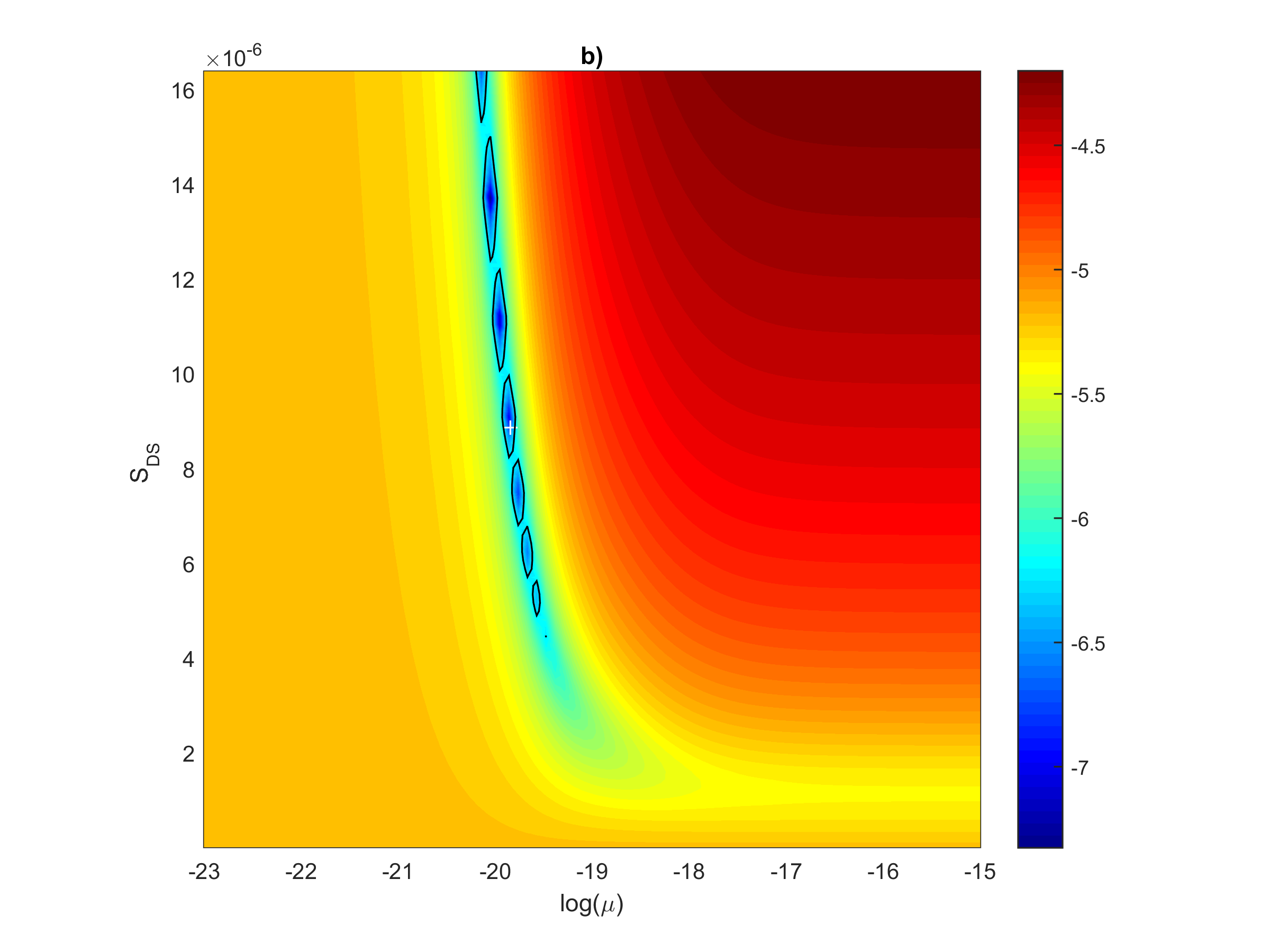

Supplement: Supplementary file 3 — Supplementary Information 3. [file 41598_2022_23493_MOESM3_ESM.zip › moment_vs_time/SD23/4_2/nm3xxx/p3.tif]

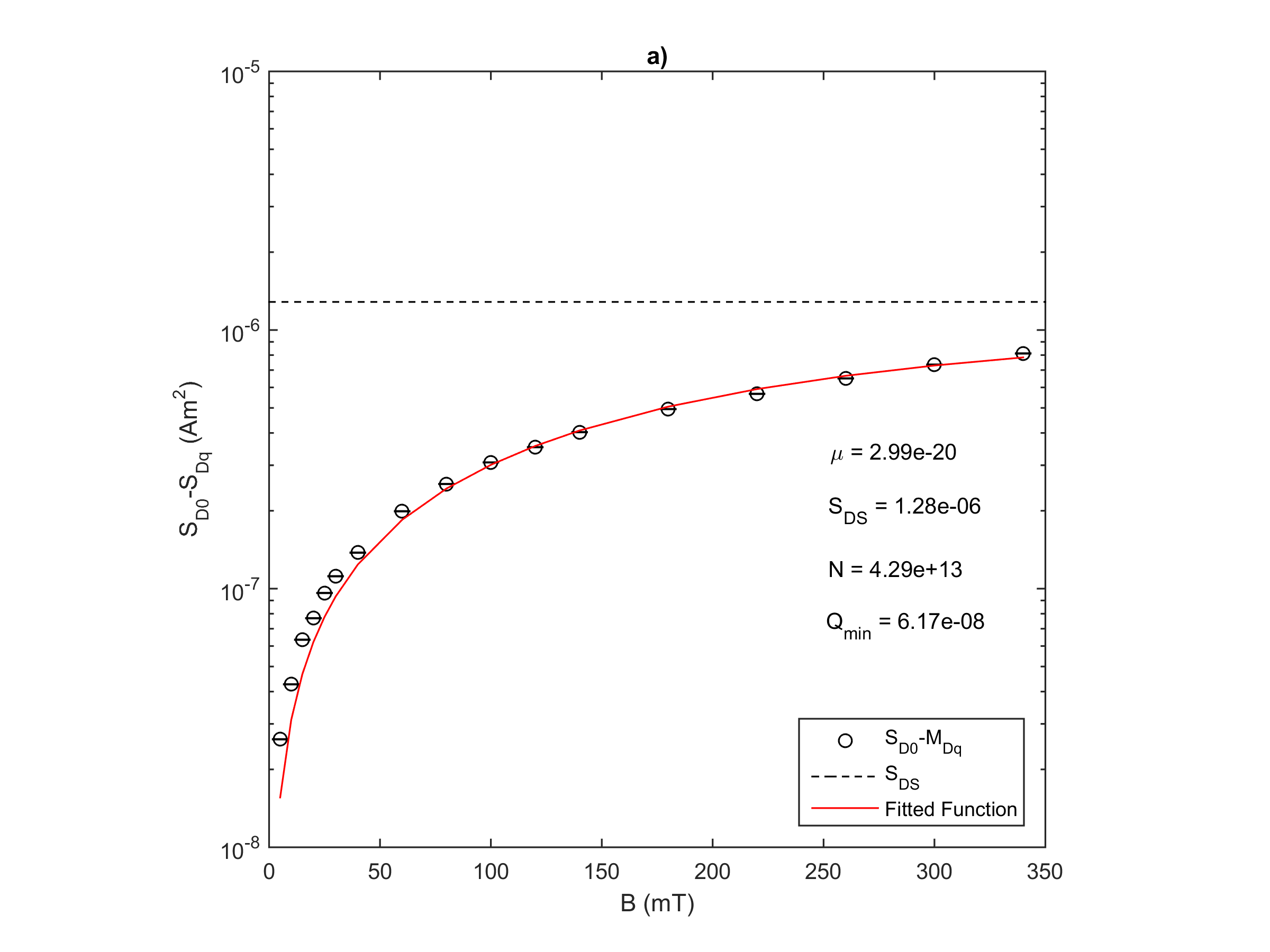

Supplement: Supplementary file 3 — Supplementary Information 3. [file 41598_2022_23493_MOESM3_ESM.zip › moment_vs_time/SD23/5_1/nm4xxx/ajuste.png]

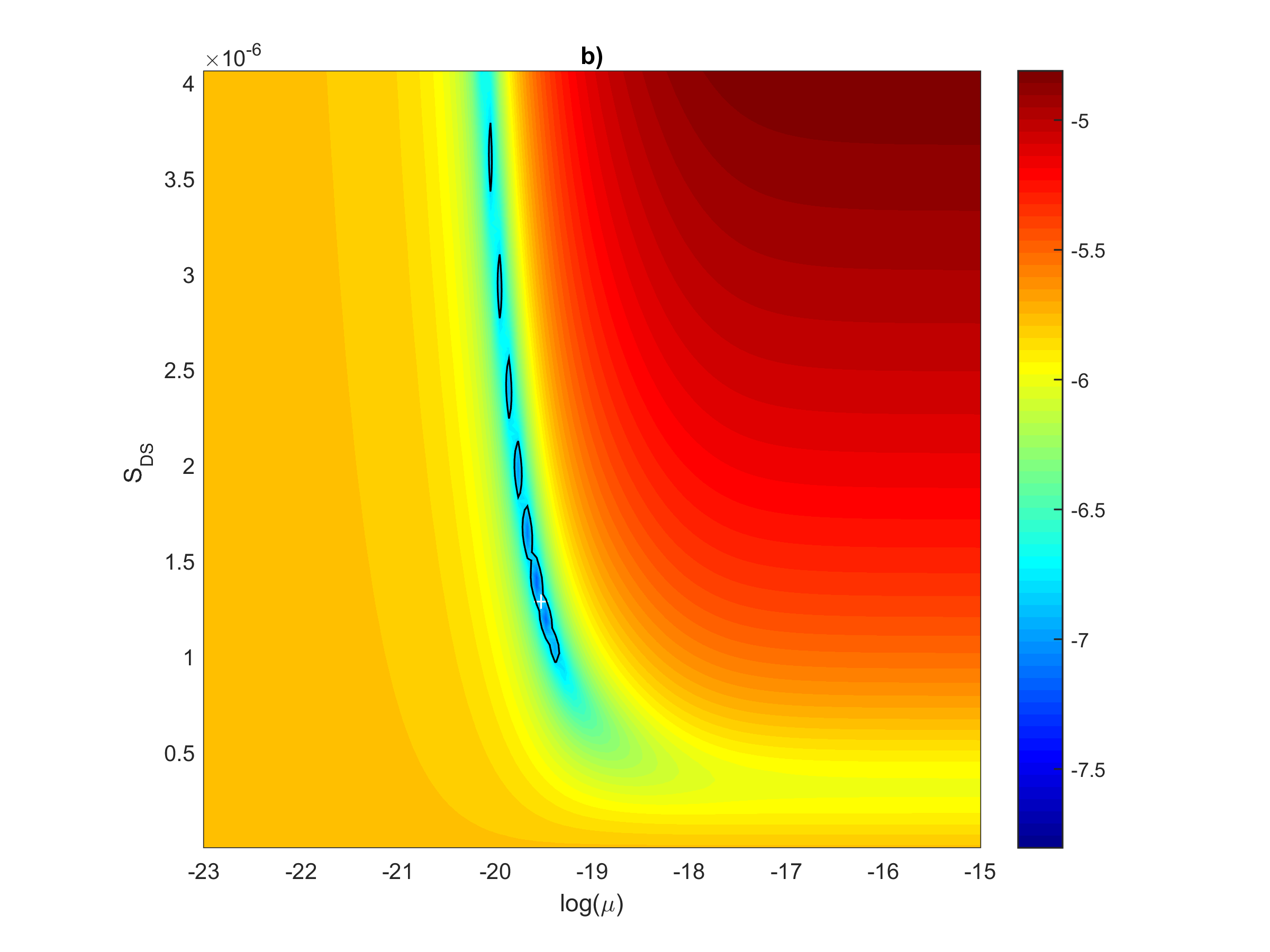

Supplement: Supplementary file 3 — Supplementary Information 3. [file 41598_2022_23493_MOESM3_ESM.zip › moment_vs_time/SD23/5_1/nm4xxx/mapa.png]

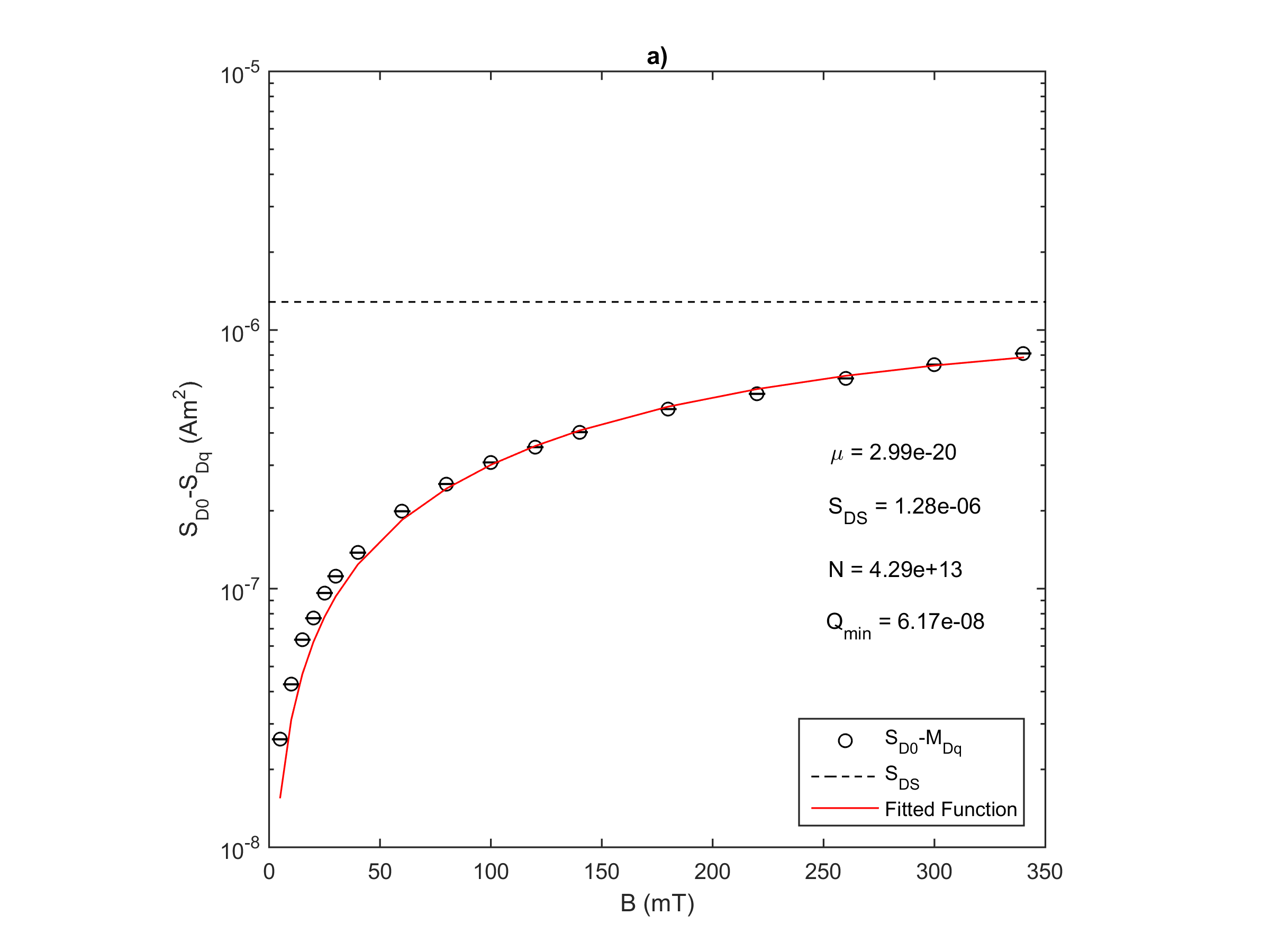

Supplement: Supplementary file 3 — Supplementary Information 3. [file 41598_2022_23493_MOESM3_ESM.zip › moment_vs_time/SD23/5_1/nm4xxx/p2.tif]

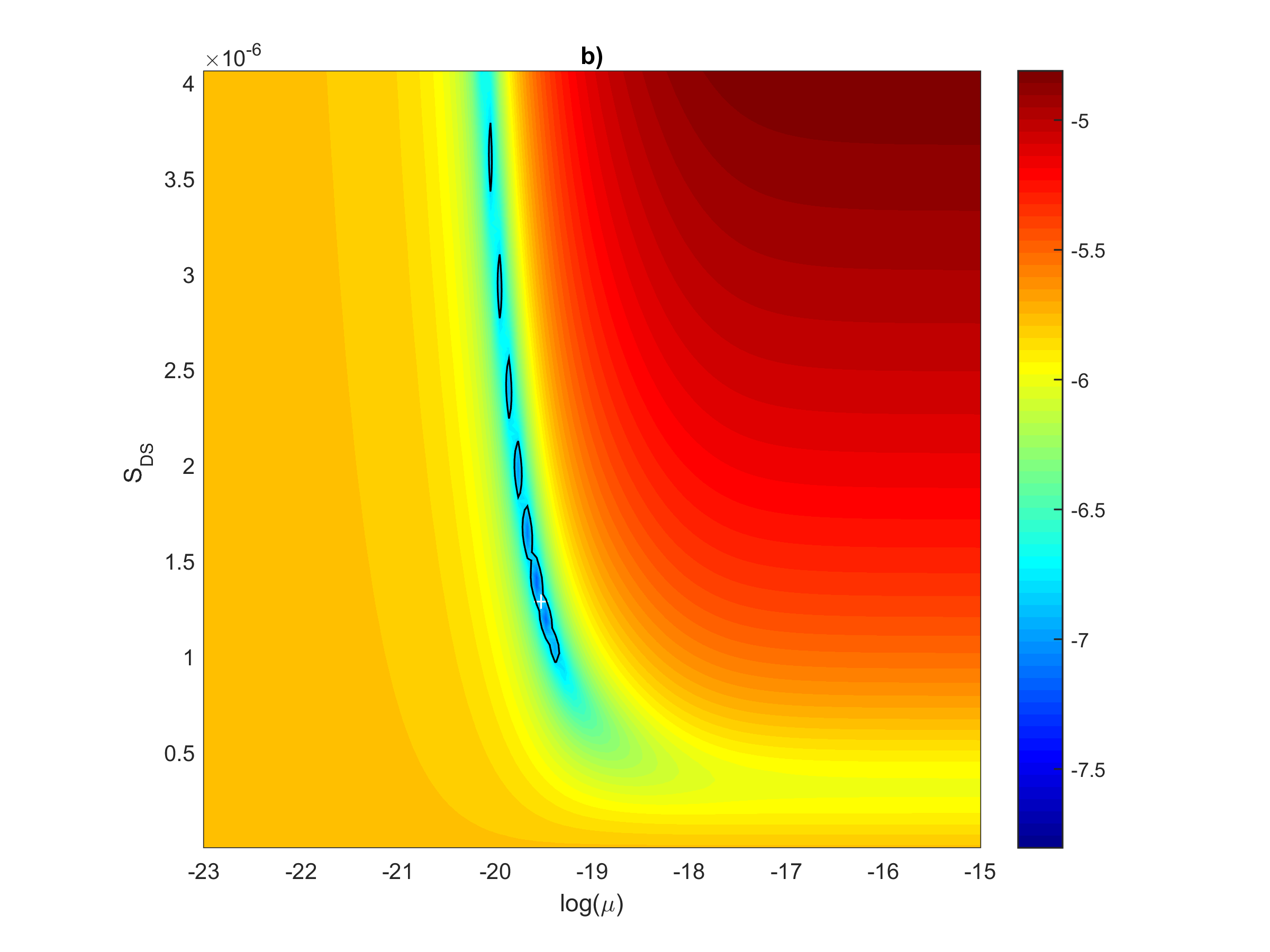

Supplement: Supplementary file 3 — Supplementary Information 3. [file 41598_2022_23493_MOESM3_ESM.zip › moment_vs_time/SD23/5_1/nm4xxx/p3.tif]

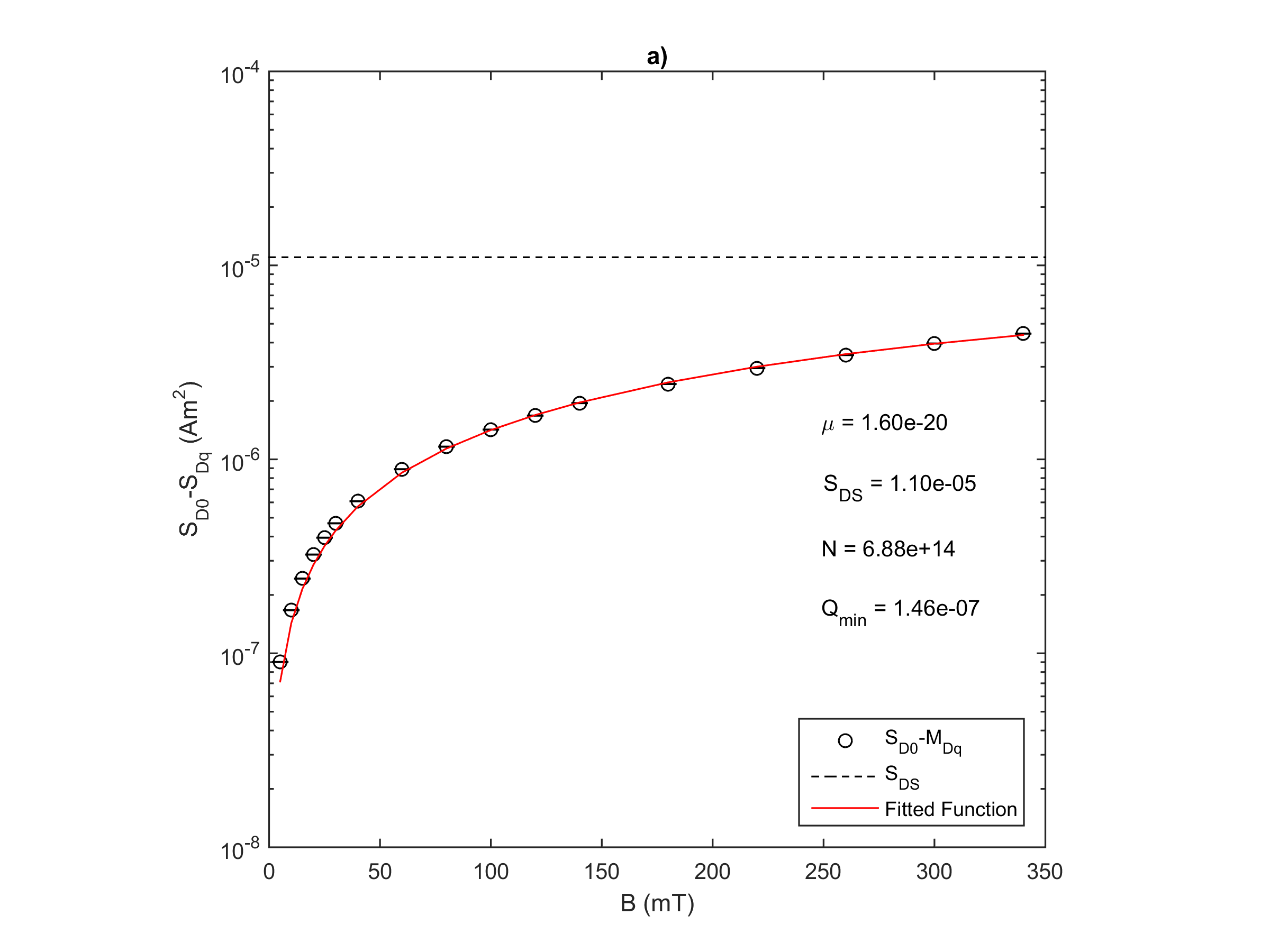

Supplement: Supplementary file 3 — Supplementary Information 3. [file 41598_2022_23493_MOESM3_ESM.zip › moment_vs_time/SD34/2_7/ajuste.png]

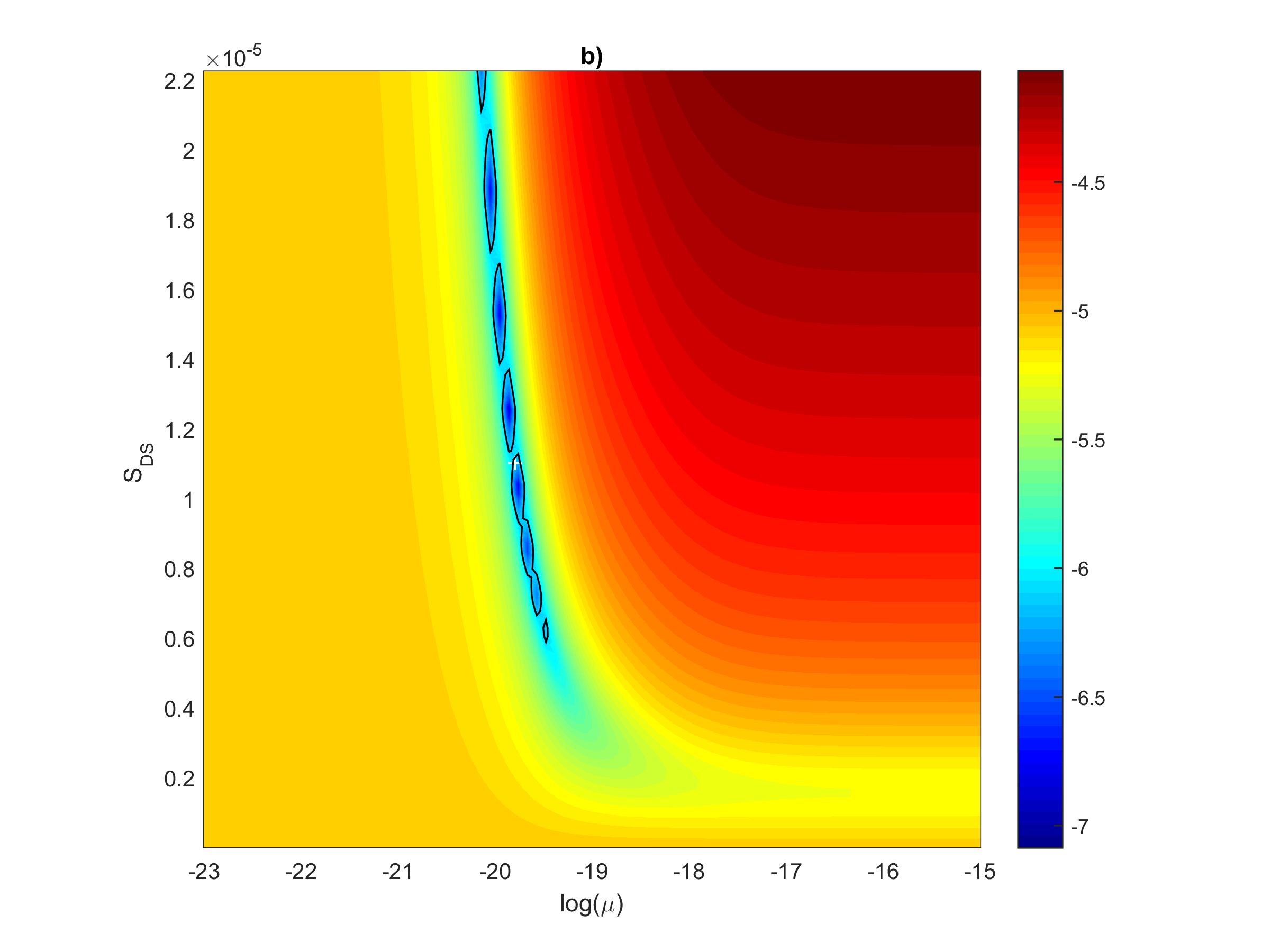

Supplement: Supplementary file 3 — Supplementary Information 3. [file 41598_2022_23493_MOESM3_ESM.zip › moment_vs_time/SD34/2_7/mapa.png]

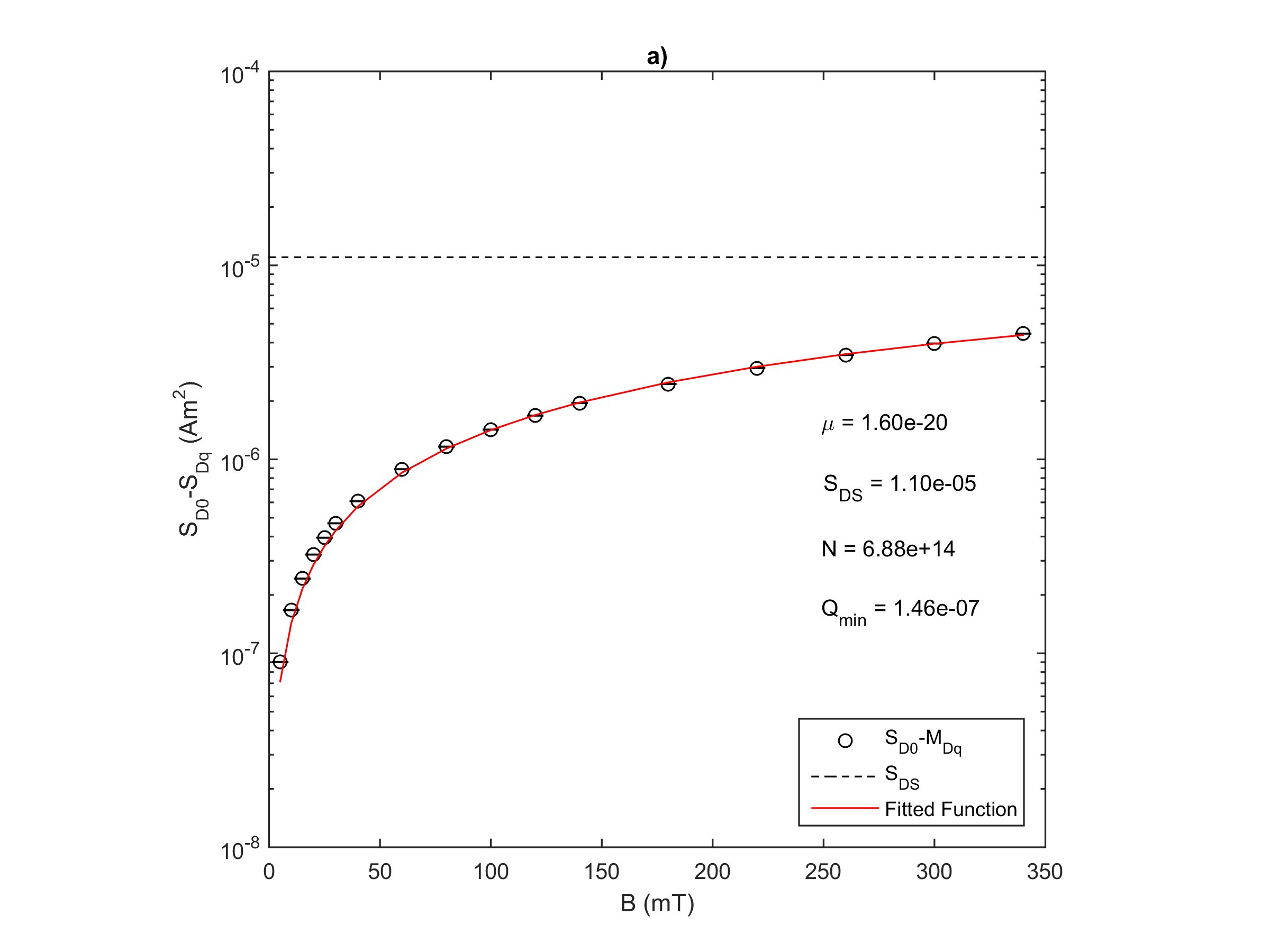

Supplement: Supplementary file 3 — Supplementary Information 3. [file 41598_2022_23493_MOESM3_ESM.zip › moment_vs_time/SD34/2_7/p2.tif]

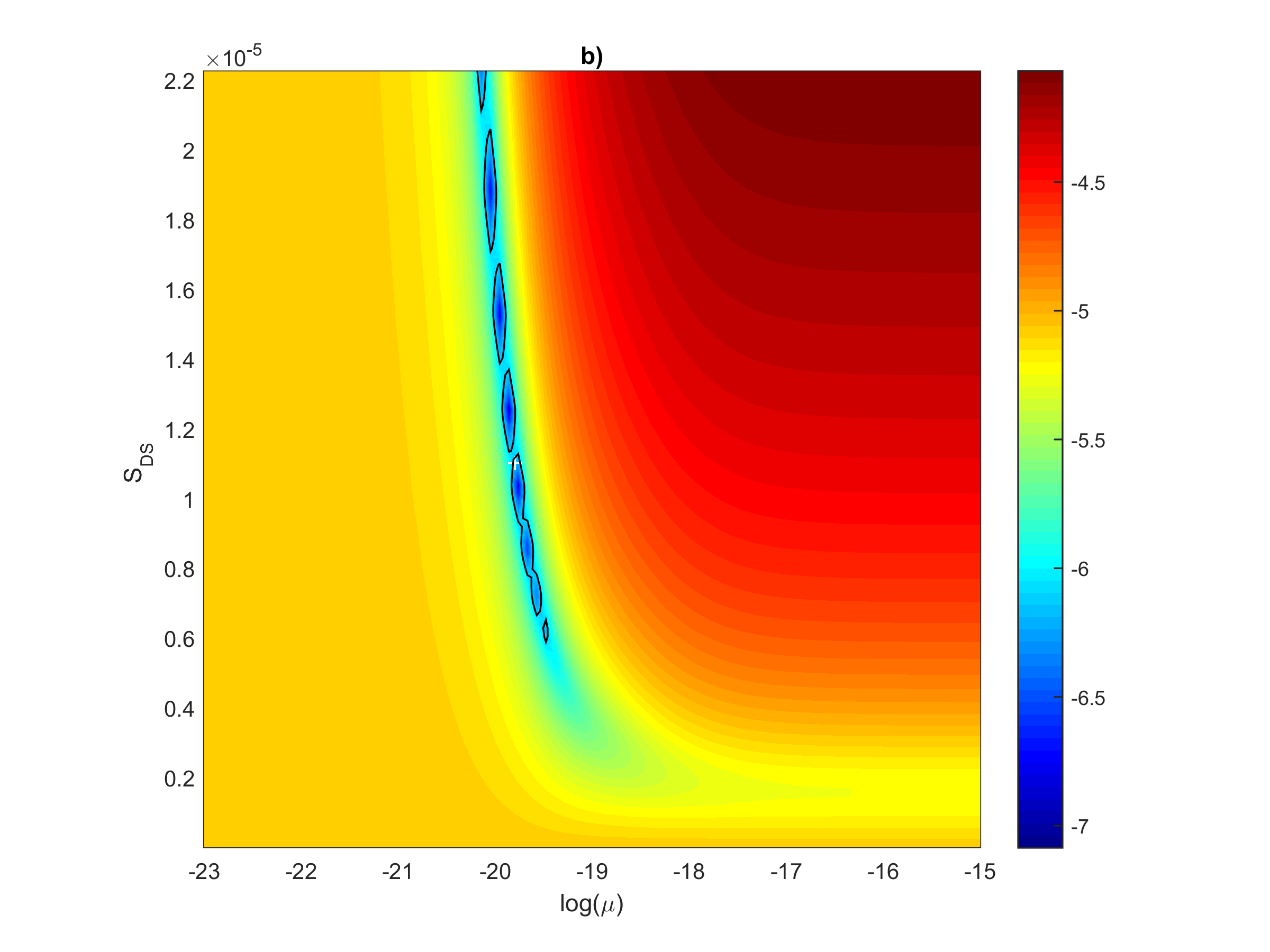

Supplement: Supplementary file 3 — Supplementary Information 3. [file 41598_2022_23493_MOESM3_ESM.zip › moment_vs_time/SD34/2_7/p3.tif]

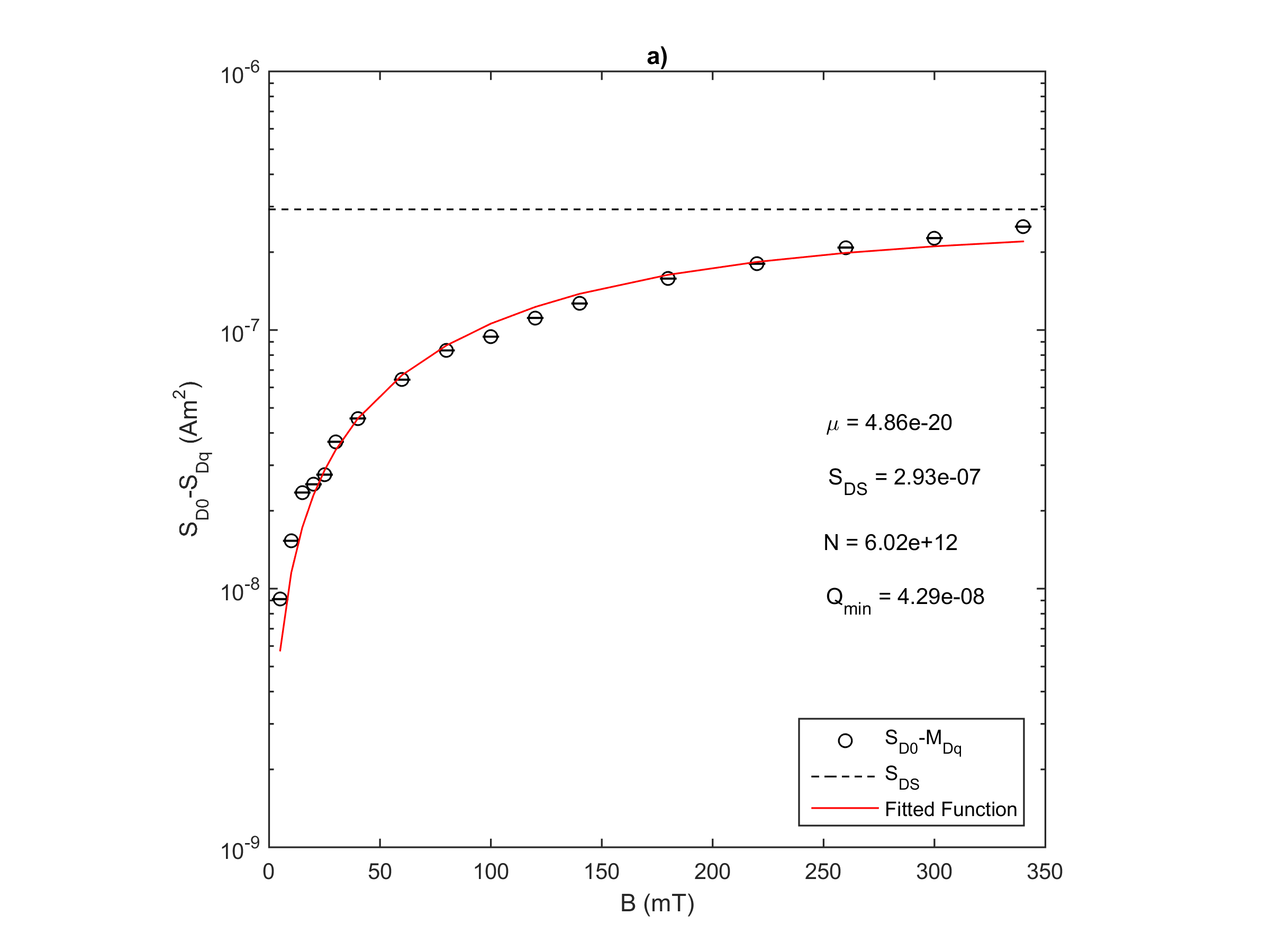

Supplement: Supplementary file 3 — Supplementary Information 3. [file 41598_2022_23493_MOESM3_ESM.zip › moment_vs_time/SD34/4_2/nm2xxx/ajuste.png]

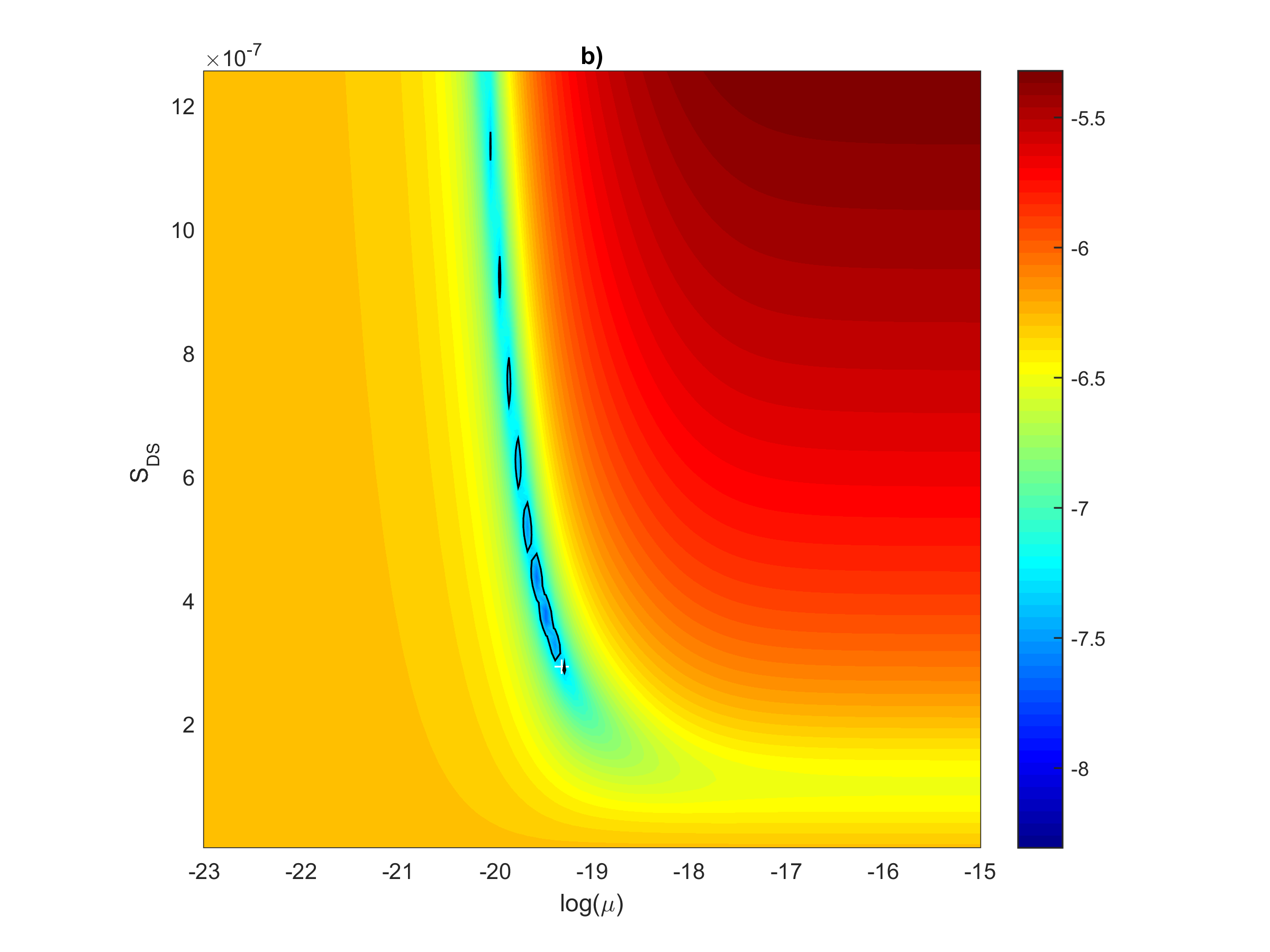

Supplement: Supplementary file 3 — Supplementary Information 3. [file 41598_2022_23493_MOESM3_ESM.zip › moment_vs_time/SD34/4_2/nm2xxx/mapa.png]

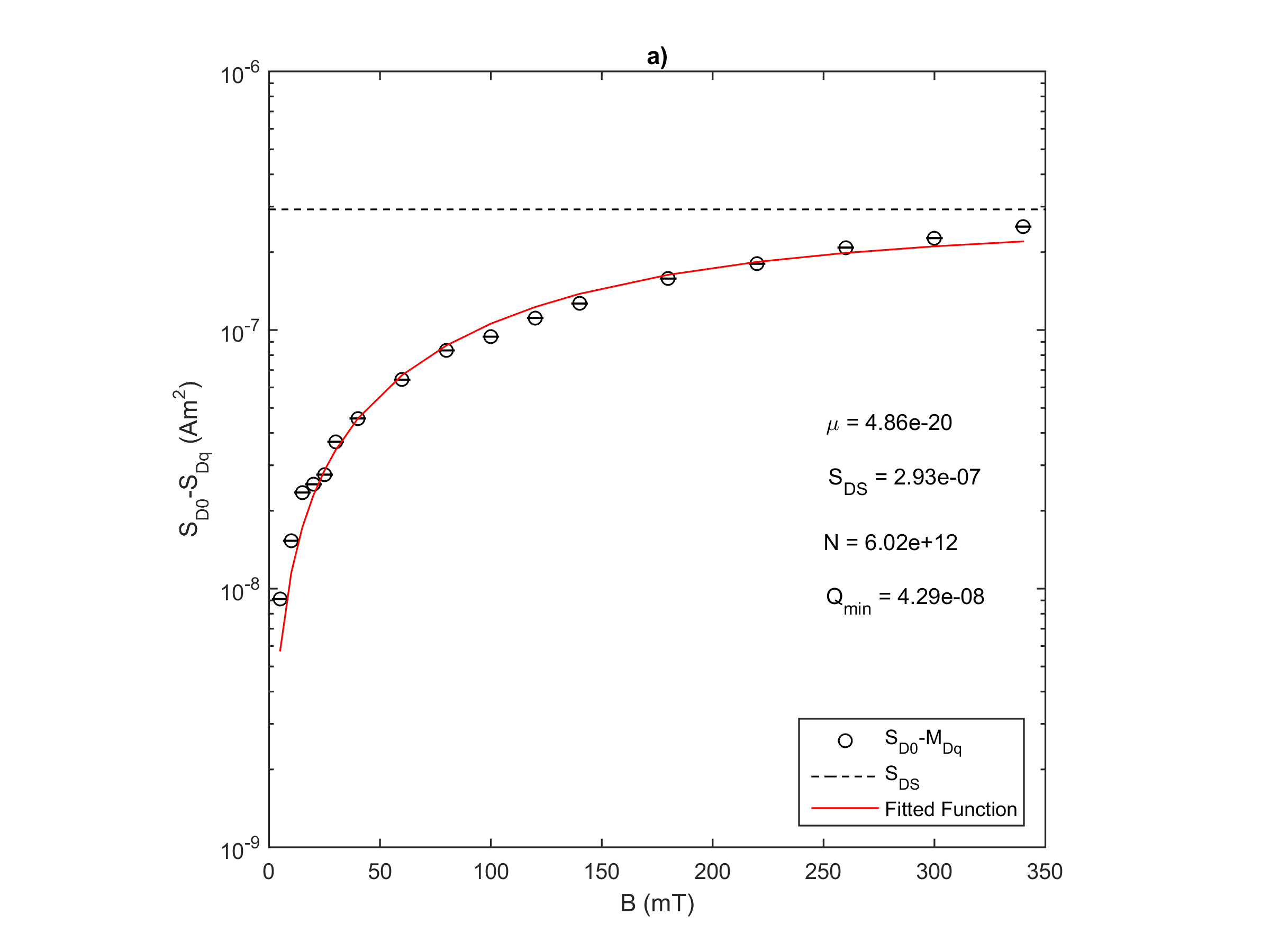

Supplement: Supplementary file 3 — Supplementary Information 3. [file 41598_2022_23493_MOESM3_ESM.zip › moment_vs_time/SD34/4_2/nm2xxx/p2.tif]

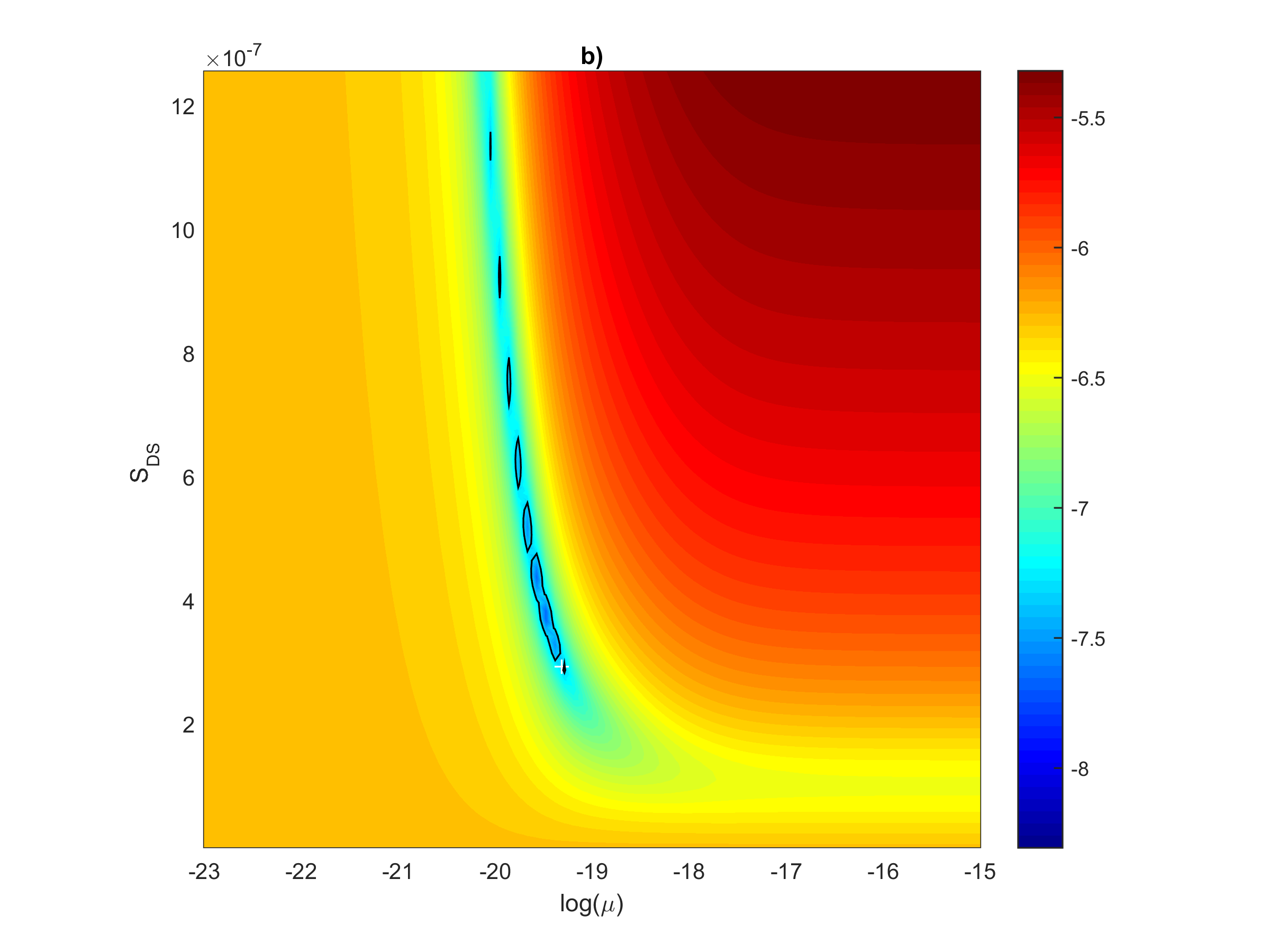

Supplement: Supplementary file 3 — Supplementary Information 3. [file 41598_2022_23493_MOESM3_ESM.zip › moment_vs_time/SD34/4_2/nm2xxx/p3.tif]

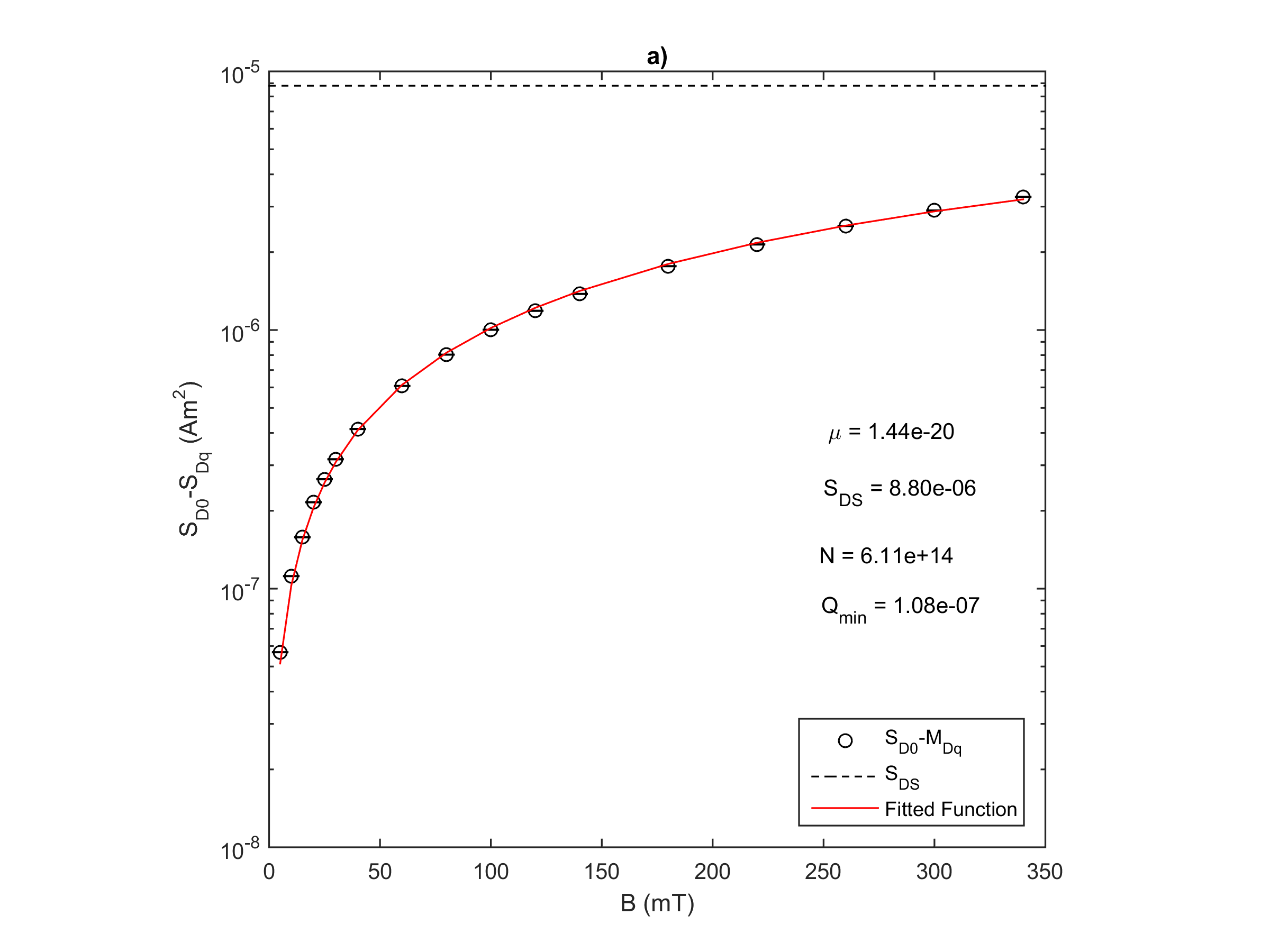

Supplement: Supplementary file 3 — Supplementary Information 3. [file 41598_2022_23493_MOESM3_ESM.zip › moment_vs_time/SD34/4_8/nm3xxx/ajuste.png]

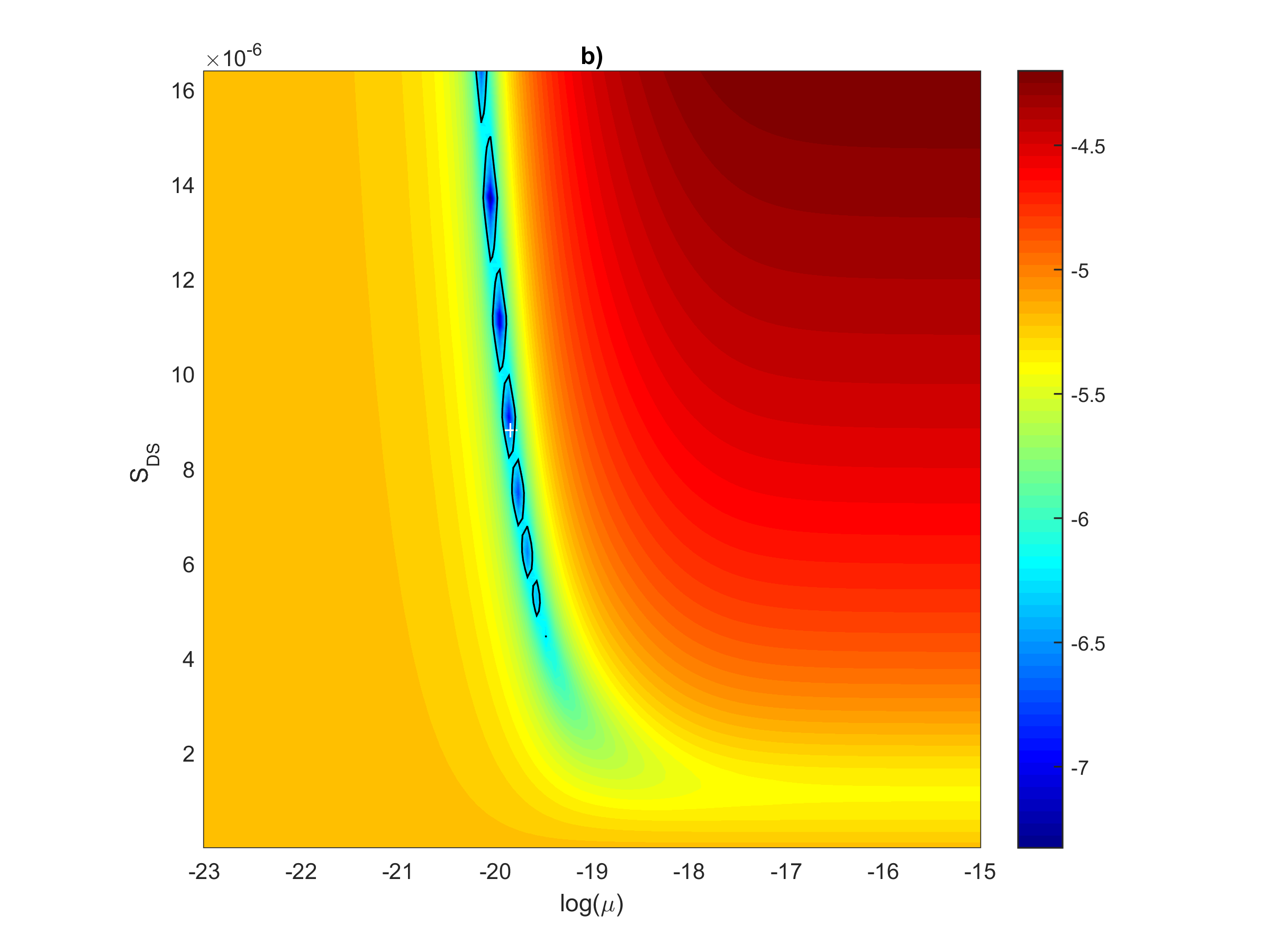

Supplement: Supplementary file 3 — Supplementary Information 3. [file 41598_2022_23493_MOESM3_ESM.zip › moment_vs_time/SD34/4_8/nm3xxx/mapa.png]

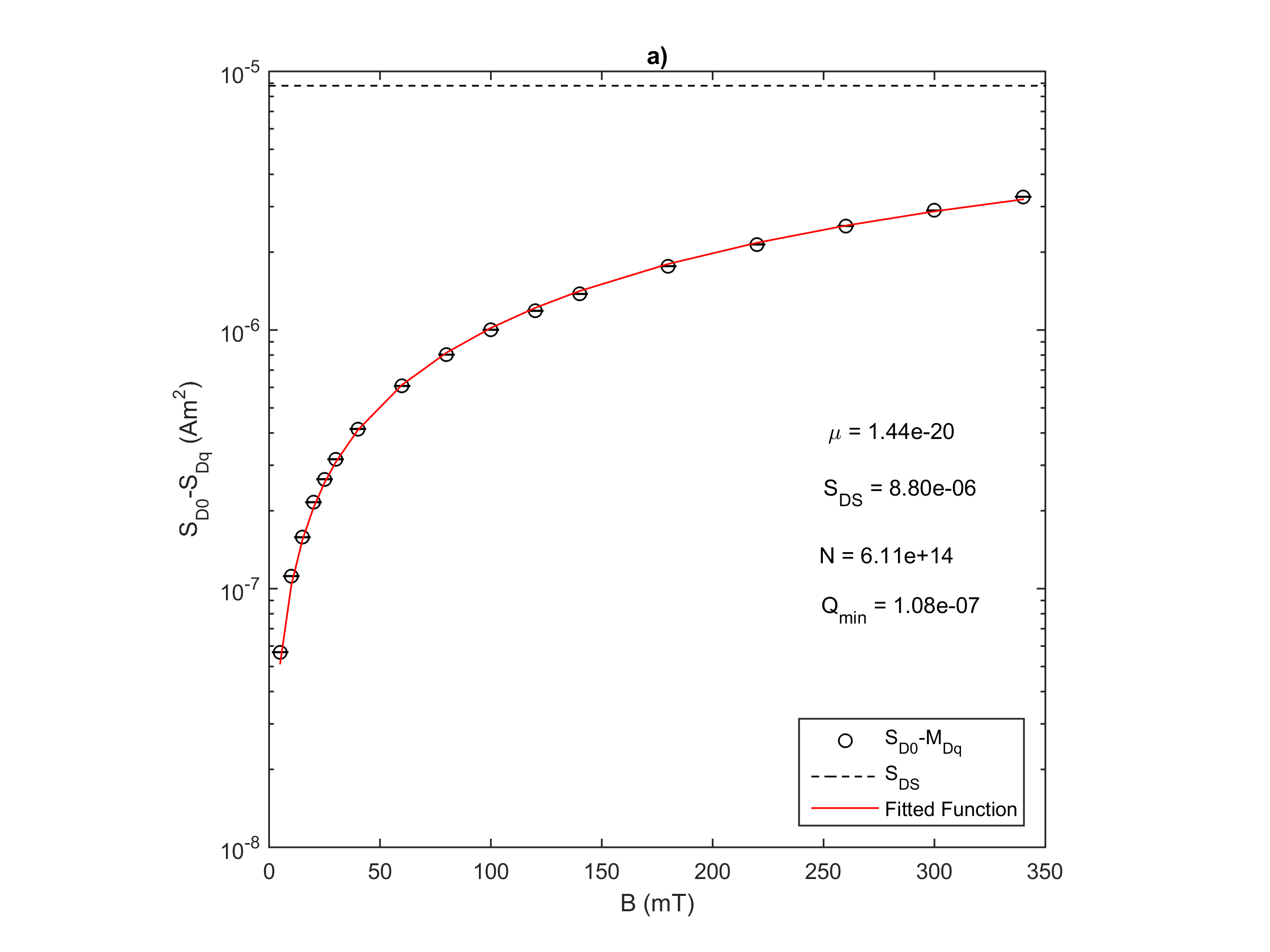

Supplement: Supplementary file 3 — Supplementary Information 3. [file 41598_2022_23493_MOESM3_ESM.zip › moment_vs_time/SD34/4_8/nm3xxx/p2.tif]

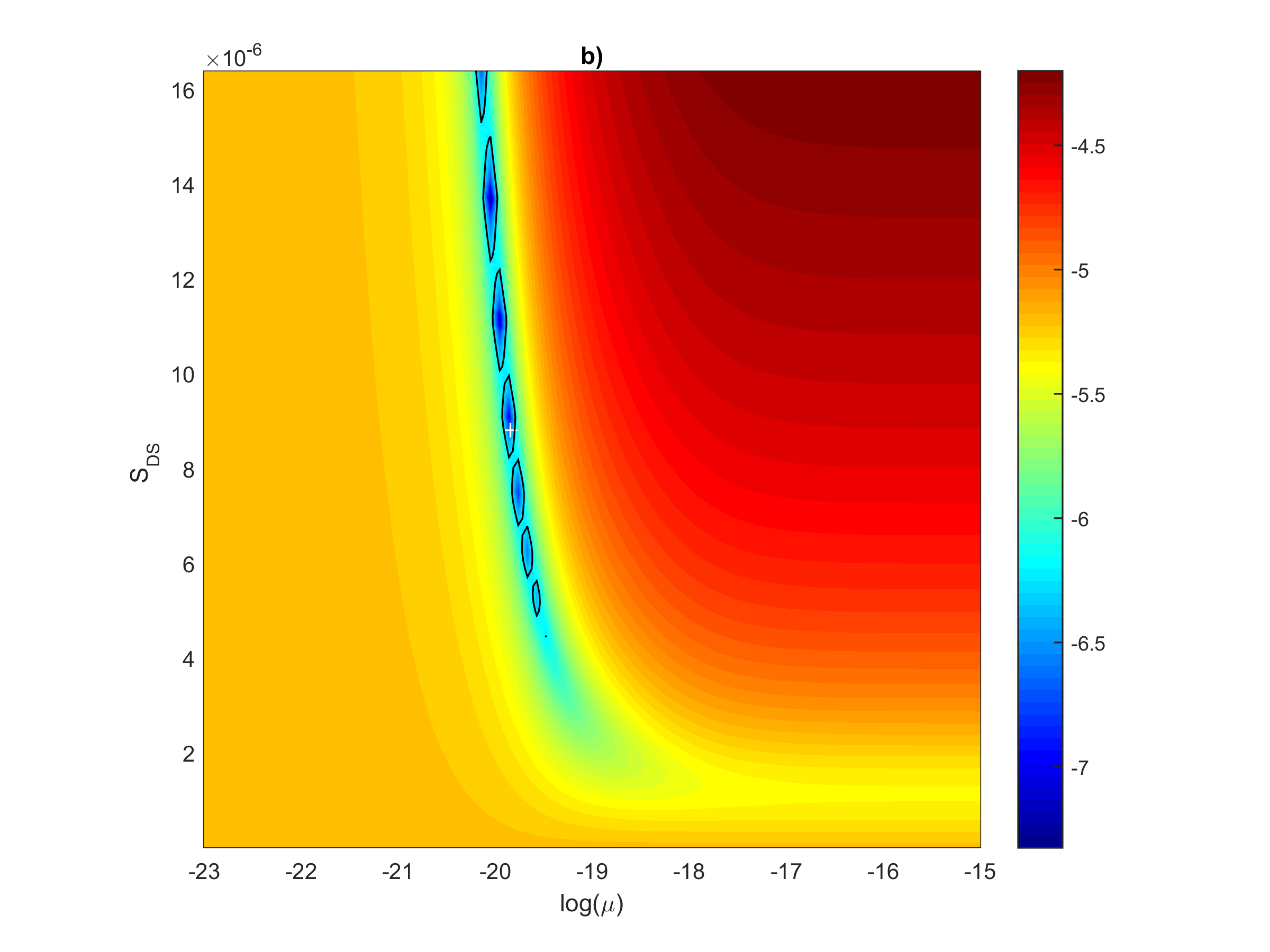

Supplement: Supplementary file 3 — Supplementary Information 3. [file 41598_2022_23493_MOESM3_ESM.zip › moment_vs_time/SD34/4_8/nm3xxx/p3.tif]

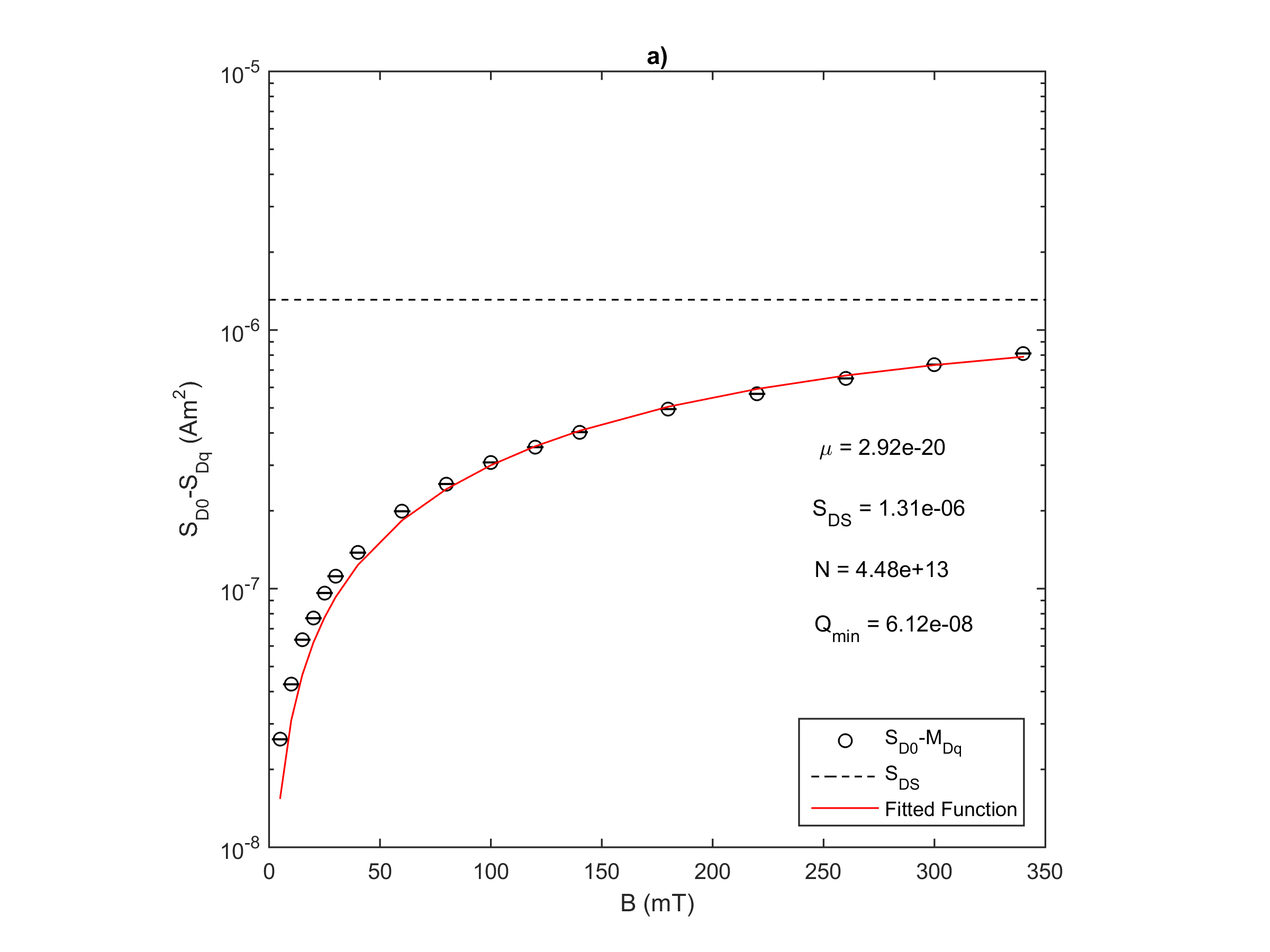

Supplement: Supplementary file 3 — Supplementary Information 3. [file 41598_2022_23493_MOESM3_ESM.zip › moment_vs_time/SD34/5_7/nm4xxx/ajuste.png]

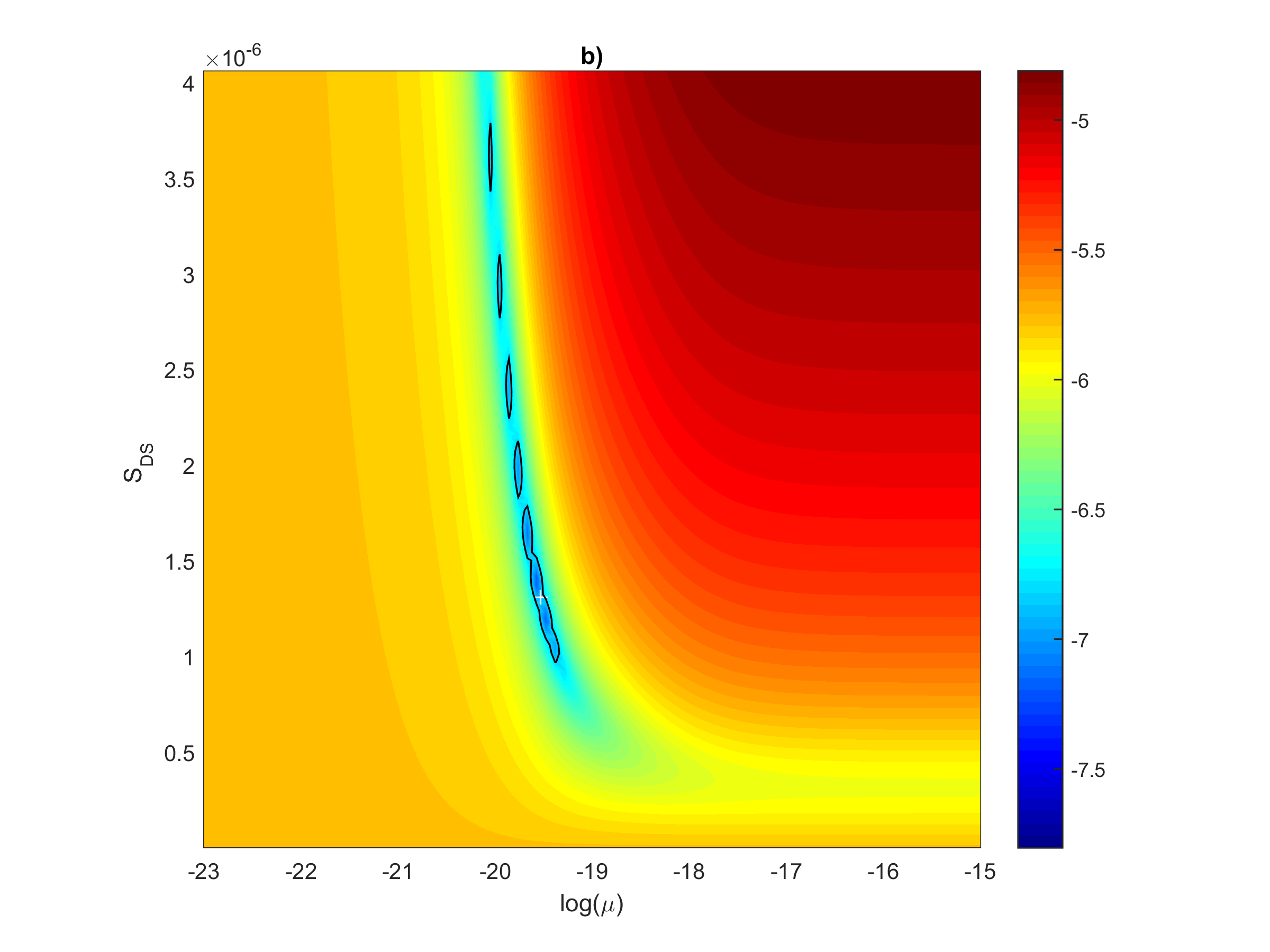

Supplement: Supplementary file 3 — Supplementary Information 3. [file 41598_2022_23493_MOESM3_ESM.zip › moment_vs_time/SD34/5_7/nm4xxx/mapa.png]

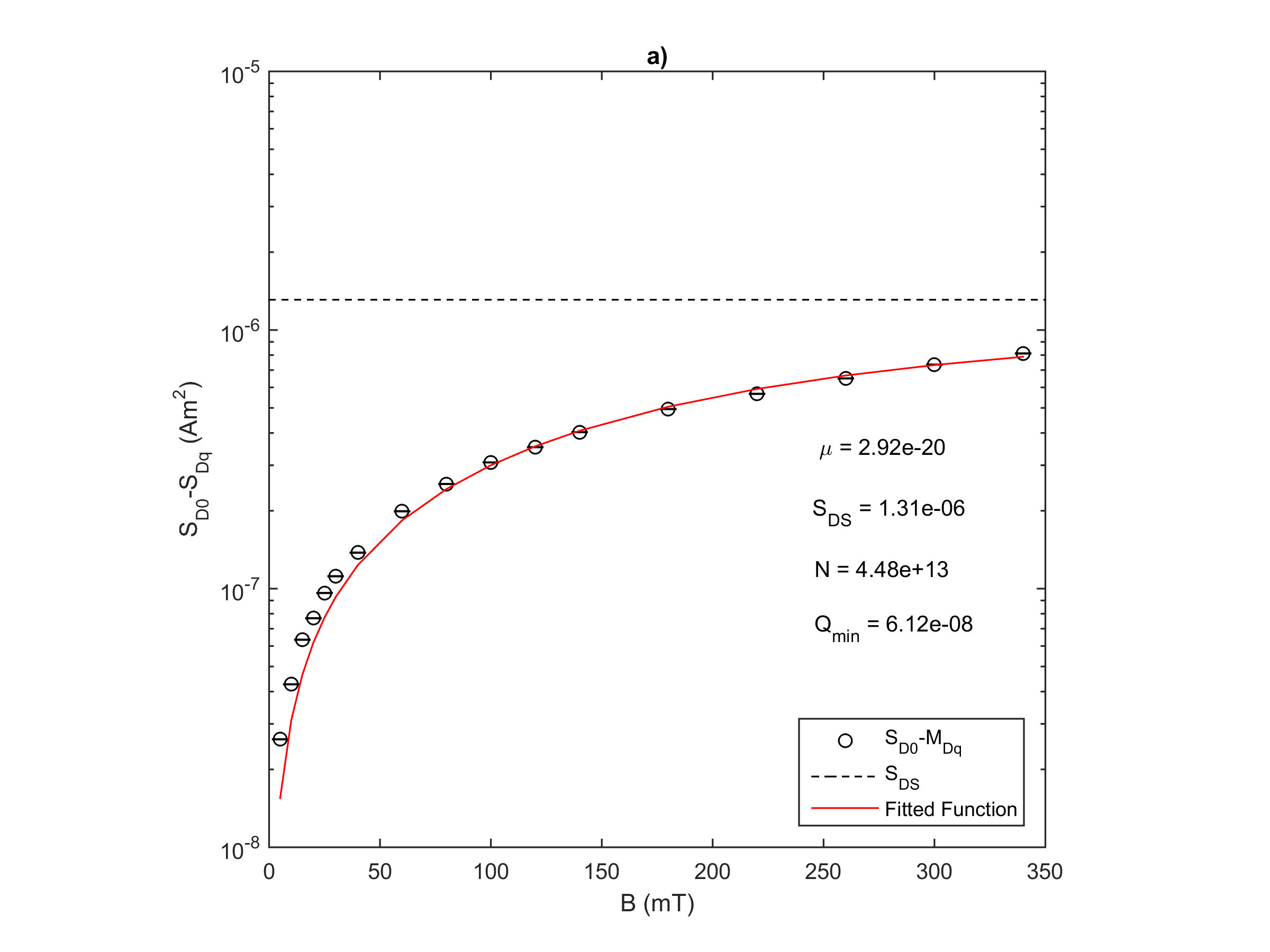

Supplement: Supplementary file 3 — Supplementary Information 3. [file 41598_2022_23493_MOESM3_ESM.zip › moment_vs_time/SD34/5_7/nm4xxx/p2.tif]

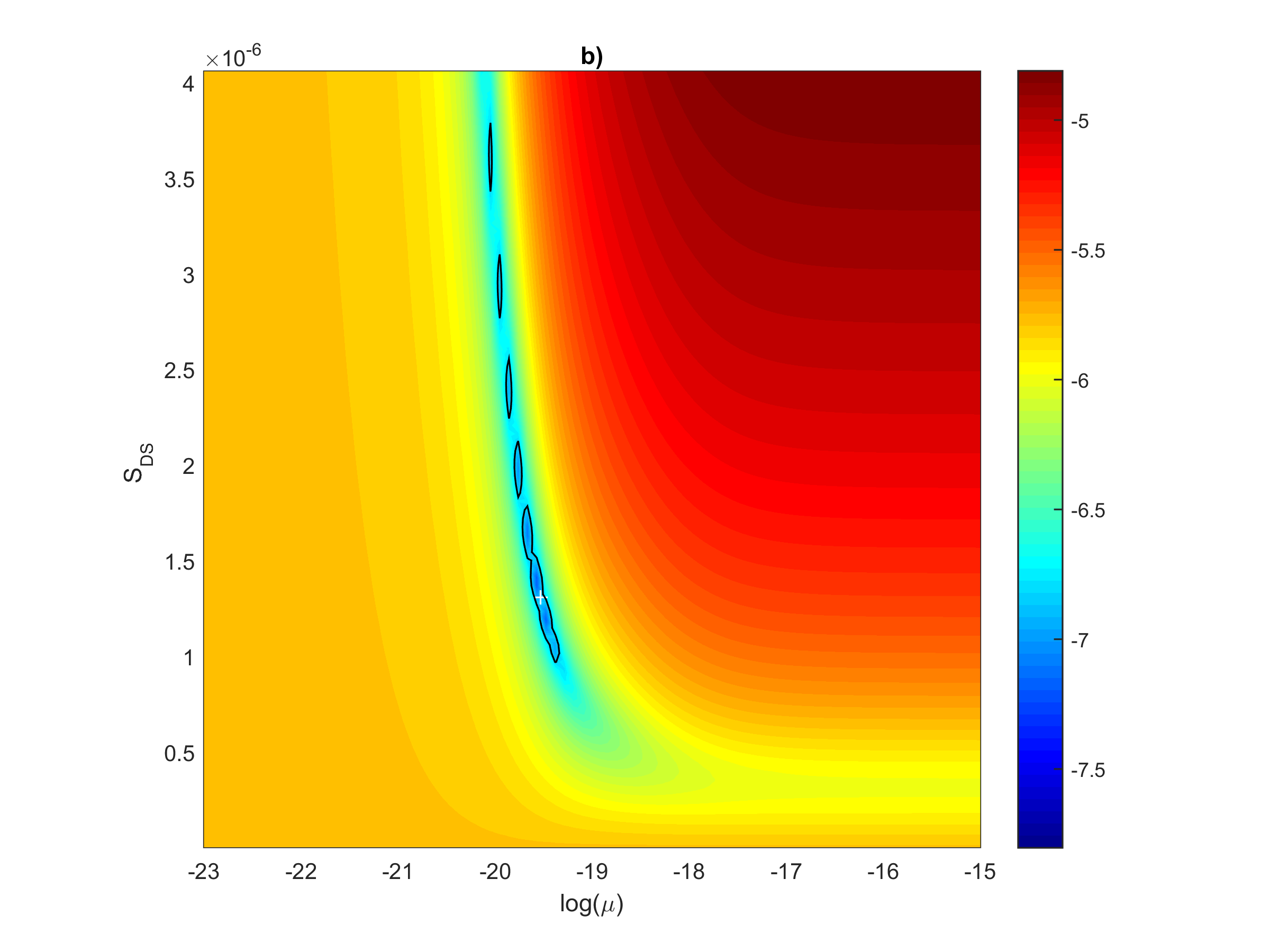

Supplement: Supplementary file 3 — Supplementary Information 3. [file 41598_2022_23493_MOESM3_ESM.zip › moment_vs_time/SD34/5_7/nm4xxx/p3.tif]

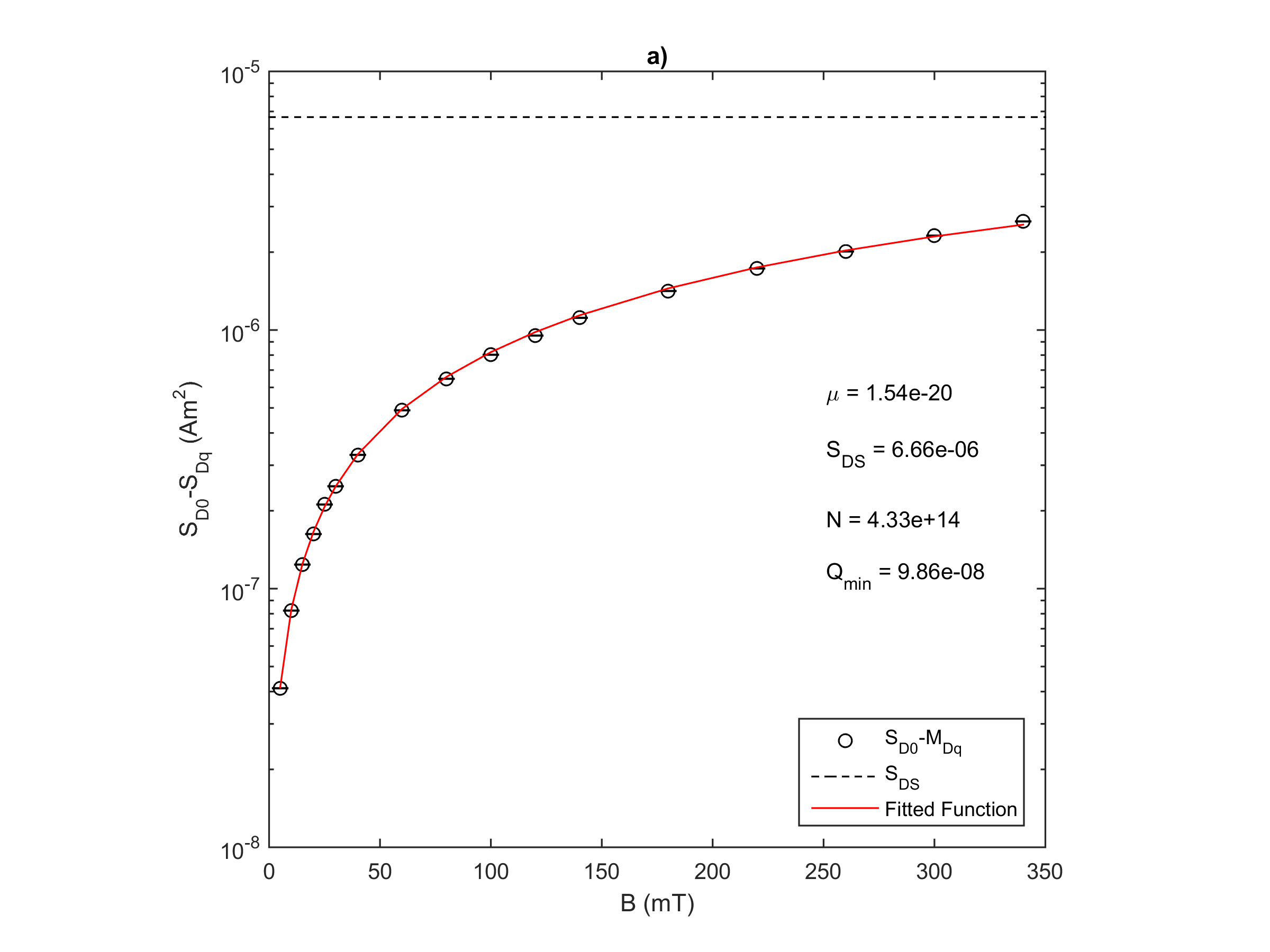

Supplement: Supplementary file 3 — Supplementary Information 3. [file 41598_2022_23493_MOESM3_ESM.zip › moment_vs_time/SD35/2_35/nm1xxx/ajuste.png]

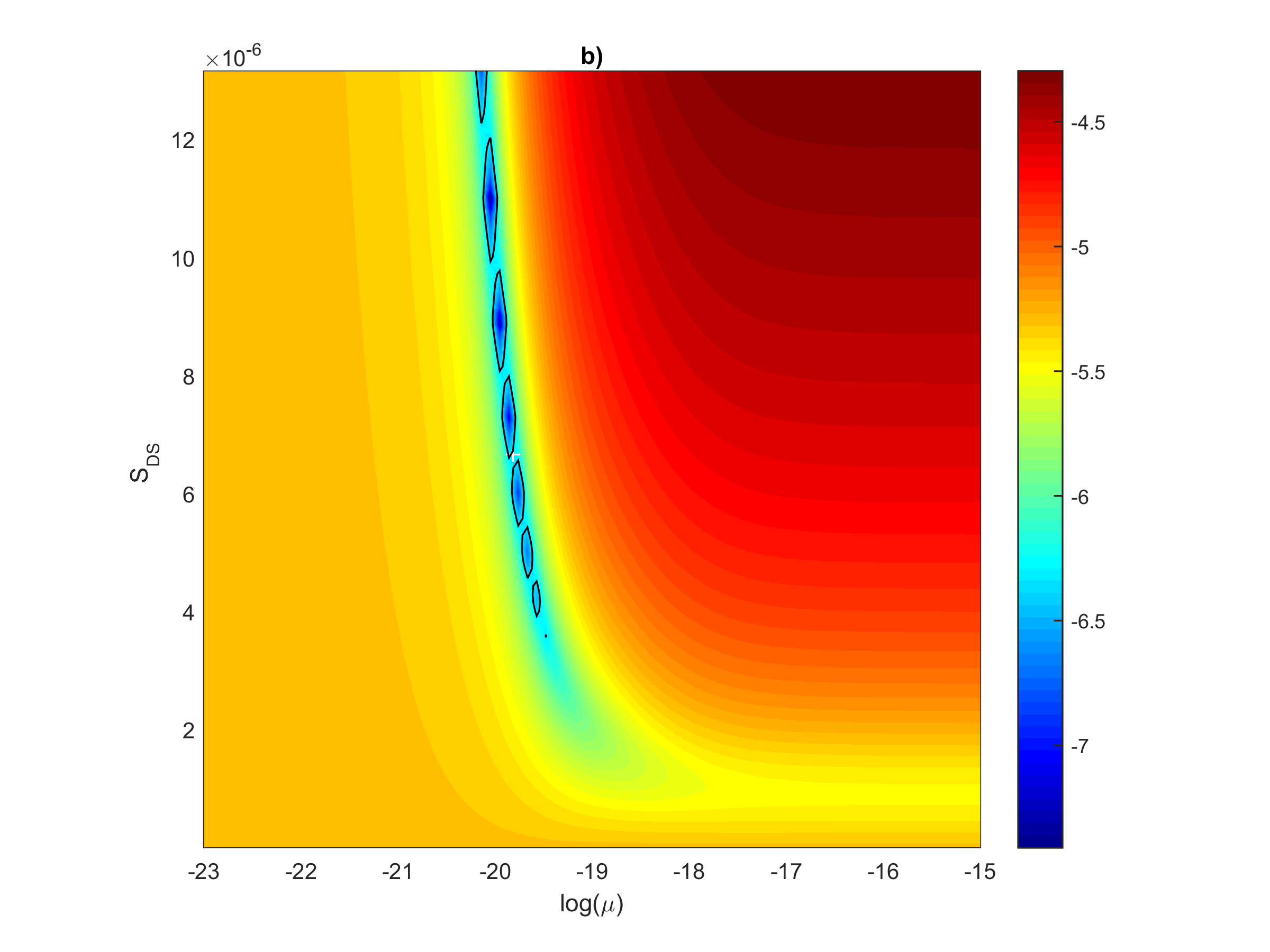

Supplement: Supplementary file 3 — Supplementary Information 3. [file 41598_2022_23493_MOESM3_ESM.zip › moment_vs_time/SD35/2_35/nm1xxx/mapa.png]

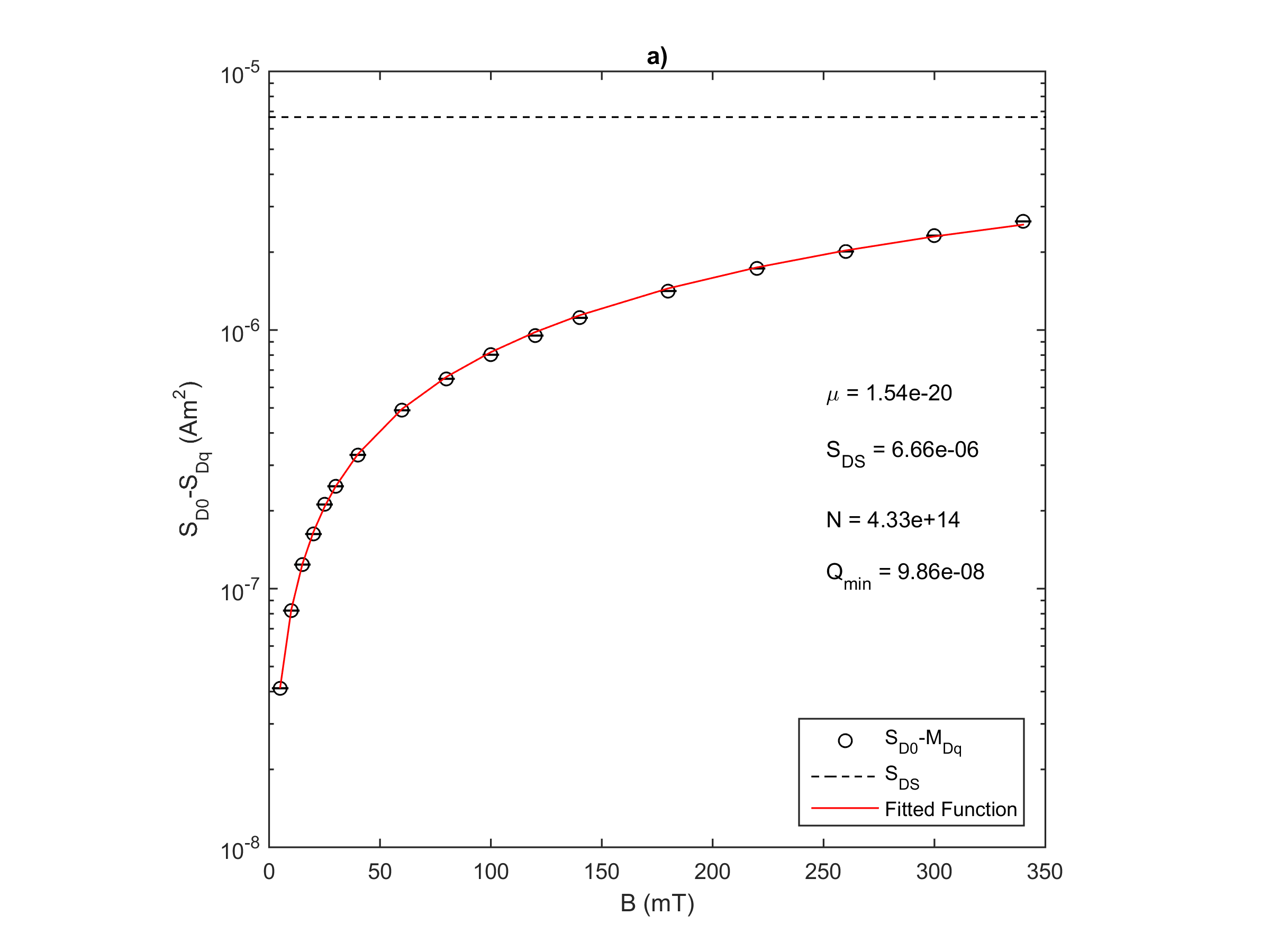

Supplement: Supplementary file 3 — Supplementary Information 3. [file 41598_2022_23493_MOESM3_ESM.zip › moment_vs_time/SD35/2_35/nm1xxx/p2.tif]

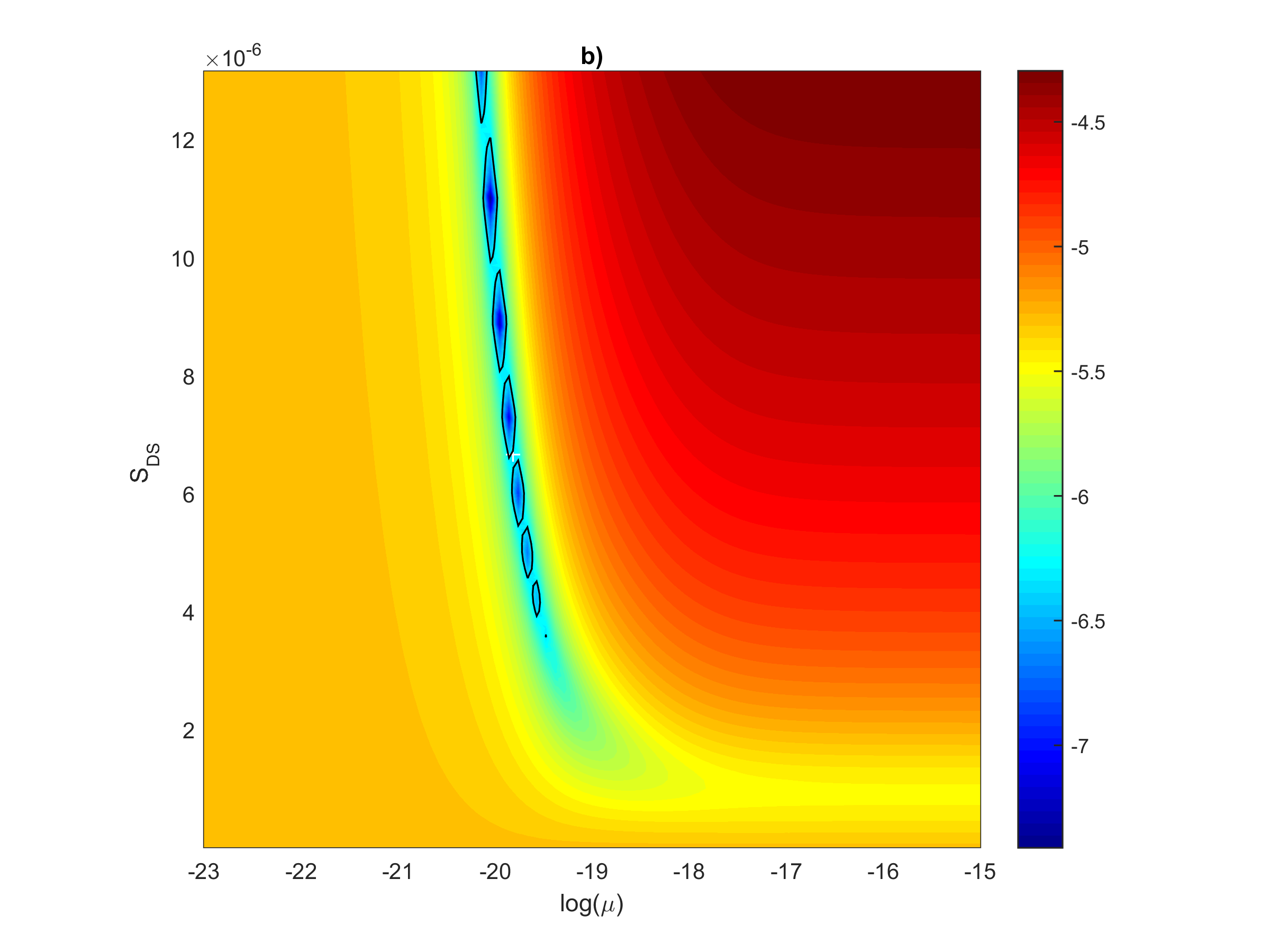

Supplement: Supplementary file 3 — Supplementary Information 3. [file 41598_2022_23493_MOESM3_ESM.zip › moment_vs_time/SD35/2_35/nm1xxx/p3.tif]
